# The Small GTPase Arf1 Modulates Arp2/3-Mediated Actin Polymerization via PICK1 to Regulate Synaptic Plasticity

**DOI:** 10.1016/j.neuron.2013.05.003

**Published:** 2013-07-24

**Authors:** Daniel L. Rocca, Mascia Amici, Anna Antoniou, Elena Blanco Suarez, Nagaraj Halemani, Kai Murk, Jennifer McGarvey, Nadia Jaafari, Jack R. Mellor, Graham L. Collingridge, Jonathan G. Hanley

**Affiliations:** 1 School of Biochemistry, Centre for Synaptic Plasticity, Medical Sciences Building and Dorothy Hodgkin Building, University of Bristol, University Walk, Bristol BS8 1TD, UK; 2 School of Physiology and Pharmacology, Centre for Synaptic Plasticity, Medical Sciences Building and Dorothy Hodgkin Building, University of Bristol, University Walk, Bristol BS8 1TD, UK; 3 Department of Brain and Cognitive Sciences, College of Natural Sciences, Seoul National University, Seoul 151-746, Korea

## Abstract

Inhibition of Arp2/3-mediated actin polymerization by PICK1 is a central mechanism to AMPA receptor (AMPAR) internalization and long-term depression (LTD), although the signaling pathways that modulate this process in response to NMDA receptor (NMDAR) activation are unknown. Here, we define a function for the GTPase Arf1 in this process. We show that Arf1-GTP binds PICK1 to limit PICK1-mediated inhibition of Arp2/3 activity. Expression of mutant Arf1 that does not bind PICK1 leads to reduced surface levels of GluA2-containing AMPARs and smaller spines in hippocampal neurons, which occludes subsequent NMDA-induced AMPAR internalization and spine shrinkage. In organotypic slices, NMDAR-dependent LTD of AMPAR excitatory postsynaptic currents is abolished in neurons expressing mutant Arf1. Furthermore, NMDAR stimulation downregulates Arf1 activation and binding to PICK1 via the Arf-GAP GIT1. This study defines Arf1 as a critical regulator of actin dynamics and synaptic function via modulation of PICK1.

## Highlights

• The Arf1-PICK1-Arp2/3 pathway regulates actin polymerization
• NMDAR activation activates the Arf-GAP GIT1 to deactivate Arf1
• Arf1 controls NMDAR-dependent, PICK1-mediated AMPAR trafficking and LTD
• A noncanonical role is described for Arf1 in vesicle traffic, distinct from COPI regulation

## Teaser

Rocca et al. show that Arf1 regulates actin dynamics in dendritic spines by modulating PICK1-mediated Arp2/3 inhibition. This controls spine size, AMPAR trafficking, and hence synaptic transmission. This study defines Arf1 as a critical regulator of actin dynamics and synaptic plasticity via PICK1 modulation.

## Introduction

Long-term synaptic plasticity is thought to underlie learning and memory and is also important for the fine-tuning of neural circuitry during development. AMPA receptors (AMPARs) mediate the majority of fast excitatory synaptic transmission in the brain, and plasticity at excitatory synapses involves alterations in AMPAR number at the synaptic plasma membrane in processes involving the regulated trafficking of AMPAR-containing vesicles (Collingridge et al., 2010; Shepherd and Huganir, 2007).

The dynamic actin cytoskeleton is central to the regulation of vesicle trafficking by exerting mechanical forces that alter membrane geometry (Kaksonen et al., 2006). Localized alterations in actin turnover are proposed to provide mechanical forces that contribute to membrane curvature, vesicle scission, and propulsion of nascent vesicles away from the membrane (Merrifield, 2004). The molecular machinery and upstream signaling pathways that regulate actin polymerization are therefore of fundamental importance to the control of receptor trafficking and their expression on the cell surface. The Arp2/3 complex is the major catalyst for the formation of branched actin networks that mediate membrane remodelling (Pollard, 2007; Stradal and Scita, 2006). Dendritic spines are the sites of excitatory synapses in neurons and are particularly enriched in extremely dynamic filamentous F-actin, which cycles rapidly between F-actin and globular G-actin (Star et al., 2002). The dynamic actin cytoskeleton plays a crucial role in the regulation of AMPAR trafficking that underlies synaptic plasticity (Cingolani and Goda, 2008); however, the mechanisms that regulate actin polymerization to control AMPAR trafficking during synaptic plasticity are not well understood.

PICK1 is a PDZ- and BAR-domain-containing protein that binds, via the PDZ domain, to AMPAR subunits GluA2/3 (Hanley, 2008; Xu and Xia, 2006). PICK1 is required for AMPAR internalization in response to Ca^2+^ influx via NMDA receptor (NMDAR) activation in hippocampal neurons, which underlies the reduction in synaptic strength in long-term depression (LTD; Hanley and Henley, 2005; Terashima et al., 2008; Volk et al., 2010). PICK1-mediated GluA2 trafficking is also a crucial mechanism in cerebellar LTD (Steinberg et al., 2006; Xia et al., 2000), indicating the central importance of PICK1 in synaptic plasticity. We recently demonstrated that PICK1 directly binds to and inhibits the actin-nucleating Arp2/3 complex and that this plays a central role in AMPAR trafficking, spine shrinkage, and LTD in hippocampal neurons (Nakamura et al., 2011; Rocca et al., 2008). How NMDAR activation modulates PICK1-mediated Arp2/3 inhibition to trigger changes in AMPAR trafficking and spine dynamics is unknown. A number of proteins regulating Arp2/3 activity, such as N-WASP and WAVE, are effectors for the small GTPases Cdc42 and Rac, respectively, and are therefore modulated by signaling pathways directed by these GTPases (Takenawa and Suetsugu, 2007). PICK1 shows homology to arfaptin, which binds the related GTPase ADP-ribosylation factor 1 (Arf1), and it has been suggested that PICK1 interacts with Arf1 in the yeast two-hybrid system (Takeya et al., 2000). The functional consequences of this interaction are completely unexplored.

The Arf proteins are small guanosine triphosphate (GTP)-binding proteins that are typically associated with trafficking of membrane proteins. Arfs promote vesicle biogenesis by recruiting coat protein complexes such as COPI to the sites of vesicle formation (D’Souza-Schorey and Chavrier, 2006; Gillingham and Munro, 2007). More recently, it has become apparent that Arfs can regulate actin cytoskeleton dynamics as part of this membrane trafficking process (Dubois et al., 2005; Myers and Casanova, 2008), although the molecular mechanisms remain unclear, especially in neurons.

In this study, we demonstrate that PICK1 is an Arf1 effector, whereby Arf1 signaling modulates the inhibition of Arp2/3-mediated actin polymerization by PICK1 in dendritic spines. Via its interaction with PICK1, Arf1 regulates spine size and the trafficking of GluA2-containing AMPARs in hippocampal neurons. Furthermore, we identify an NMDAR-mediated pathway involving GIT1, Arf1, and PICK1 that regulates synaptic function and LTD.

## Results

### GTP-Bound Arf1 Binds Directly to PICK1 and Forms a Triple Complex with GluA2 in Neurons

**Figure 1:**
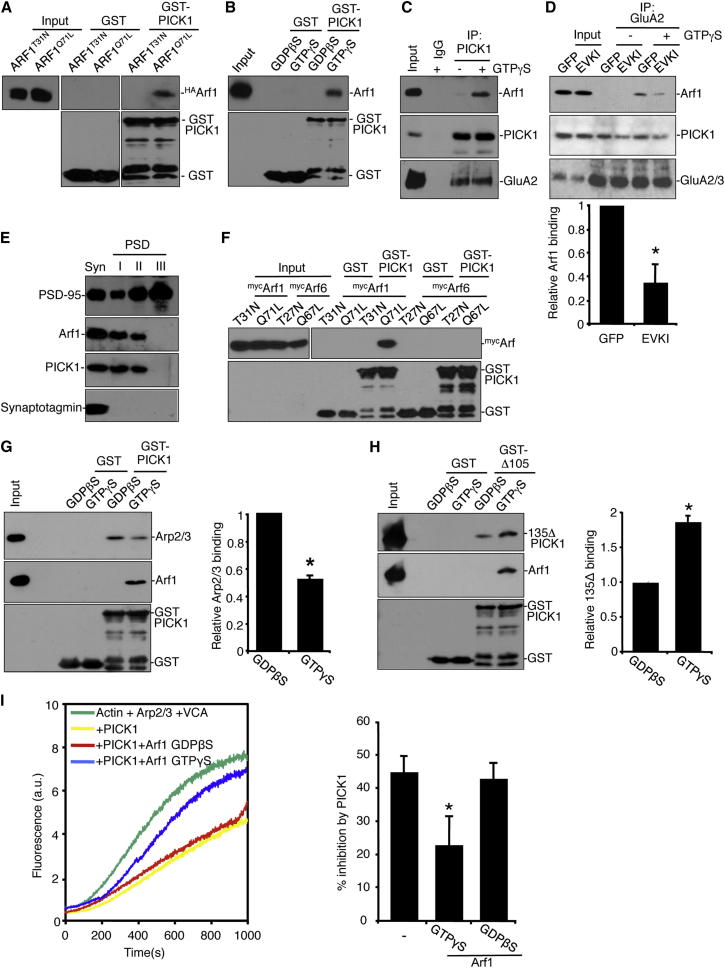
Activated Arf1 Binds PICK1 to Block Arp2/3 Inhibition (A) GST-PICK1 interacts with constitutively active Arf1. Extracts from COS7 cells expressing dominant-negative (T31N) or constitutively active (Q71L) ^HA^Arf1 were incubated with GST-PICK1 or GST. Bound proteins were detected by western blotting using anti-HA and anti-GST. (B) Arf1 binds PICK1 directly in a GTP-dependent manner. Purified his_6_Arf1 was incubated with GST or GST-PICK1 ± 0.2 mM GTPγS. Bound proteins were detected by western blotting using anti-Arf1 and anti-GST. (C) PICK1 interacts with GTP-bound Arf1 in neurons. Cultured neurons were lysed ±GTPγS and extracts were immunoprecipitated with anti-PICK1 or control IgG. Bound proteins were detected by western blotting using antibodies as shown. (D) Arf1 interacts with GluA2 via PICK1. Extracts from cultured neurons infected with Sindbis virus expressing either GFP or pep2-EVKI were immunoprecipitated with anti-GluA2 ± GTPγS. Bound proteins were detected by western blotting using specific antibodies as shown. Association of Arf1 with the GluA2 complex is dramatically reduced when PICK1-GluA2 binding is inhibited. Graph shows quantification of the relative binding of Arf1 to the GluA2 complex in the presence of GTPγS. n = 4; values are mean ± SEM; ^∗^p < 0.01. (E) Arf1 and PICK1 are similarly associated with the postsynaptic density (PSD). PSD fractions (PSD I, II, and III) were prepared by extraction from synaptosomes (Syn). Proteins were detected by western blotting using antibodies as shown. (F) PICK1 does not bind Arf6. Extracts from COS7 cells expressing either dominant-negative (T31N) or constitutively active (Q71L) ^myc^Arf1 and either dominant-negative (T27N) or constitutively active (Q67L) ^myc^Arf6 were incubated with GST-PICK1 or GST. Bound proteins were detected by western blotting using anti-myc and anti-GST. (G) GTP-bound Arf1 inhibits the PICK1-Arp2/3 interaction. Immobilized GST-PICK1 was incubated with 10 nM purified Arp2/3 complex and either GDPβS- or GTPγS- loaded his_6_Arf1. Bound proteins were detected by western blotting. Graph shows quantification of Arp2/3 binding to GST-PICK1. n = 5; values are mean ± SEM; ^∗^p < 0.03. (H) Arf1 promotes BAR-PDZ intramolecular interactions in PICK1. GST-Δ105-PICK1 (PDZ domain deleted) complexed with his_6_135Δ-PICK1 (PDZ domain only) was immobilized on beads and incubated with either GDPβS- or GTPγS-loaded Arf1. Bound proteins were detected by western blotting. Graph shows quantification of 135Δ PICK1 bound to Δ105 PICK1. n = 3; values are mean ± SEM; ^∗^p < 0.01. (I) Activated Arf1 blocks the inhibition of VCA and Arp2/3-mediated actin polymerization by PICK1. In vitro polymerization of 2.5 μM pyrene-actin in the presence of 25 nM Arp2/3, 100 nM GST-VCA, 500 nM his_6_PICK1, and 200 nM GDPβS- or GTPγS-loaded his_6_Arf1 as shown. Shown are example traces (left) and quantification as percent inhibition by PICK1 at 50% maximal polymerization in the presence of GDP- or GTP-bound Arf1 (right). n = 4; values are mean ± SEM; ^∗^p < 0.02. See also Figure S1.

To investigate the interaction of PICK1 with Arf1, we performed GST-PICK1 pull-down assays with a constitutively active mutant of Arf1 (Arf1Q71L) or a nucleotide-binding-defective mutant (Arf1T31N) expressed in COS cells. GST-PICK1 interacts specifically with the constitutively active Arf1Q71L mutant, showing negligible binding to Arf1T31N, suggesting that the PICK1-Arf1 interaction is GTP dependent (Figure 1A). To test this further, we carried out GST-PICK1 pull-down assays with purified his_6_-Arf1 in the absence of other proteins and in the presence of either nonhydrolyzable GTP (GTPγS) or guanosine diphosphate (GDPβS). Arf1 binds PICK1 only in the presence of GTPγS, demonstrating a direct GTP-dependent interaction of Arf1 with PICK1 (Figure 1B). To investigate the PICK1-Arf1 interaction in native tissue, we carried out coimmunoprecipitations (co-IPs) from neuronal extracts using PICK1 antibodies in the presence or absence of GTPγS. Arf1 interacts with PICK1 only in the presence of GTPγS, demonstrating that a GTP-dependent PICK1-Arf1 complex exists in neurons (Figure 1C). The GluA2-PICK1 interaction is unaffected by the presence of GTPγS (Figure 1C and Figure S1A available online). Since a major function of PICK1 is regulating AMPAR trafficking via an interaction with the GluA2 subunit, we assessed whether PICK1 can complex with both GluA2 and Arf1 simultaneously. Co-IP from cultured neuronal extracts using anti-GluA2 antibodies demonstrates that Arf1 is in a GTP-dependent complex with GluA2 (Figure 1D). The GluA2-Arf1 complex is disrupted following transduction of neurons with Sindbis virus expressing a peptide (pep2-EVKI) that inhibits AMPAR-PICK1 interactions (Terashima et al., 2004, 2008), demonstrating that Arf1 associates with GluA2 via PICK1 (Figures 1D and S1B). To confirm AMPAR subunit specificity of this interaction, we carried out co-IP experiments from transfected HEK293 cells. Endogenous Arf1 forms a complex with PICK1^flag^ and ^myc^GluA2 but not ^myc^GluA1 (Figure S1C).

To analyze the subcellular distribution of Arf1, we carried out differential detergent fractionation of synaptosomes prepared from brain tissue. It has previously been shown that PICK1 is present in synaptosomal fractions as well as PSD fractions I and II but not the core PSD III fraction (Rocca et al., 2008; Xia et al., 1999). Arf1 shows a strikingly similar distribution to PICK1, demonstrating that both proteins are found in the same subcellular fractions, and are both loosely associated with the postsynaptic density (Figure 1E). Arf6 has also been implicated in AMPAR trafficking during LTD (Scholz et al., 2010), so we investigated whether this related protein binds PICK1. GST pull-downs demonstrate that Arf6 does not interact with PICK1 (Figure 1F). Taken together, these results demonstrate that PICK1 binds directly and specifically to Arf1 in a GTP-dependent manner and that GluA2 is present in the same complex in neurons.

### GTP-Bound Arf1 Regulates Inhibition of Arp2/3-Mediated Actin Polymerization by PICK1

We next investigated which aspect of PICK1 function is regulated by the interaction with Arf1. Since numerous small GTPases regulate actin polymerization via effector proteins, we hypothesized that Arf1 may modulate PICK1-mediated Arp2/3 inhibition. To test this hypothesis, we first investigated whether the PICK1-Arp2/3 interaction is regulated by Arf1. The addition of GTP-bound his_6_-Arf1 to PICK1-Arp2/3 complexes results in a significant reduction of Arp2/3 binding to PICK1 (Figure 1G). To confirm that this effect is specific for PICK1, we analyzed Arp2/3 binding to two other regulators of actin polymerization, cortactin and cofilin. The addition of GTP-bound his_6_Arf1 has no effect on the binding of Arp2/3 to these proteins (Figure S1D). A possible explanation for the reduced binding of Arp2/3 to PICK1 in the presence of Arf1 is that Arf1 and Arp2/3 compete for the same binding site. To test this, we performed the reverse experiment and analyzed Arf1 binding to PICK1 in the presence or absence of the Arp2/3 complex. The presence of Arp2/3 does not cause a reduction in Arf1 binding to PICK1 (Figure S1E), indicating that Arf1 does not regulate Arp2/3 binding by direct competition but rather functions via an allosteric mechanism. We also investigated whether Arf1 regulates the PICK1-actin interaction (Rocca et al., 2008). Arf1 causes a significant reduction in actin binding to PICK1 (Figure S1F). An intramolecular interaction between the PICK1 PDZ domain and BAR domain has previously been demonstrated, which inhibits the interactions of PICK1 with the Arp2/3 complex and with actin (Lu and Ziff, 2005; Rocca et al., 2008). To explore the mechanism behind Arf1 inhibition of Arp2/3 and actin binding to PICK1, we investigated whether Arf1 modulates this intramolecular interaction. Arf1-GTP enhances interactions between the PICK1 PDZ domain and BAR domain (Figure 1H). This suggests that GTP-bound Arf1 induces a “closed” conformation of PICK1, which binds Arp2/3 and actin less efficiently (Rocca et al., 2008).

These data strongly suggest that Arf1 can modulate the inhibition of Arp2/3-mediated actin polymerization by PICK1. To specifically test this hypothesis, we employed in vitro actin polymerization assays. These assays use fluorescent pyrene-conjugated actin, which exhibits increased fluorescence upon polymerization. Arp2/3-mediated actin polymerization can be stimulated by adding the verprolin/cofilin/acidic (VCA) domain of the Arp2/3 activator N-WASP. While PICK1 inhibits VCA-mediated actin polymerization as previously described (Rocca et al., 2008), the addition of GTP-bound Arf1 blocks PICK1-mediated inhibition of actin polymerization. At half-maximal polymerization, PICK1 alone causes a 44% inhibition of actin polymerization, whereas in the presence of PICK1 plus GTP-bound Arf1, actin polymerization is only inhibited by 23% (Figure 1I). In contrast, guanosine diphosphate (GDP)-bound Arf1 has no effect on PICK1 inhibition of Arp2/3 activity. This demonstrates that Arf1 can directly influence actin dynamics in vitro via PICK1 and furthermore that PICK1 is an effector of Arf1.

### Arf1 C Terminus Specifically Binds PICK1 PDZ Domain

**Figure 2:**
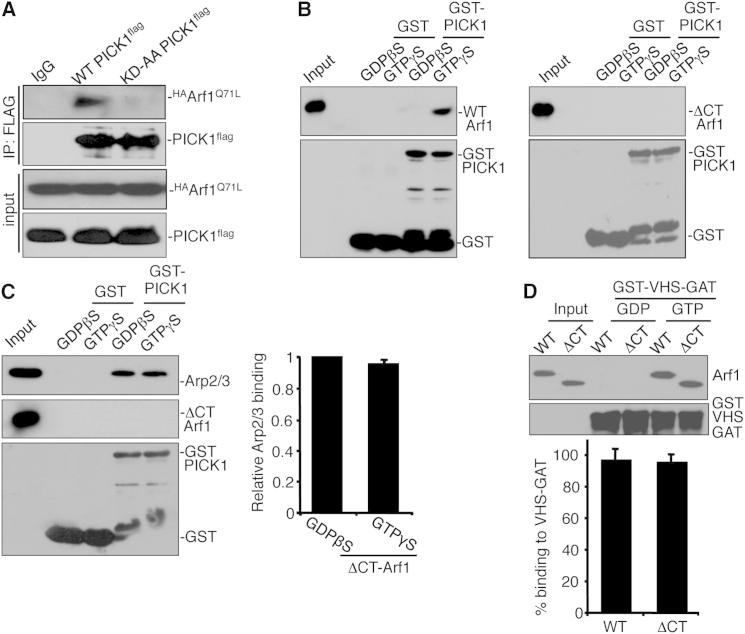
Characterization of PICK1-Arf1 Binding Mutant (A) Arf1 interacts with the PDZ domain of PICK1. COS7 cell extracts expressing WT-PICK1^FLAG^ or KD27,28AA-PICK1^FLAG^ with ^HA^Arf1^Q71L^ were immunoprecipitated with anti-FLAG or control IgG and bound proteins were detected by western blotting using anti-HA or anti-FLAG. (B) GST-PICK1 directly interacts with the C terminus of Arf1. Immobilized GST and GST-PICK1 were incubated with purified his_6_WT-Arf1 (left) or his_6_ΔCT-Arf1 (right) in the presence of either 0.2 mM GDPβS or 0.2 mM GTPγS. Bound proteins were detected by western blotting using anti-Arf1 or anti-GST. (C) ΔCT-Arf1 does not inhibit the PICK1-Arp2/3 interaction. Immobilized GST-PICK1 was incubated with purified Arp2/3 and either GDPβS- or GTPγS-bound his_6_ΔCT-Arf1. Bound proteins were detected by western blotting using anti-Arf1 or anti-GST. Graph shows quantification of Arp2/3 binding to GST-PICK1. n = 4; values are mean ± SEM. (D) The ΔCT mutation has no effect on activated Arf1 binding to the Arf1 effector protein GGA3. The immobilized GST-VHS-GAT domain of GGA3 was incubated with either GDPβS- or GTPγS-loaded his_6_WT-Arf1 and his_6_ ΔCT-Arf1. Bound proteins were detected by western blotting using anti-Arf1 or anti-GST. Graph shows quantification of percentage of Arf1 bound to GST-VHS-GAT. n = 3; values are mean ± SEM. See also Figure S2.

To investigate the binding site between Arf1 and PICK1, we carried out co-IPs from transfected COS cells and found that a mutation in the PICK1 PDZ domain (KD27,28AA; Terashima et al., 2004) abolishes the interaction with Arf1 (Figure 2A). This is consistent with yeast two-hybrid data in a previous report, which also suggested that PICK1 interacts with the C terminus of Arf1 (Takeya et al., 2000). We show that in GST pull-down assays, deletion of the extreme C-terminal four amino acids on Arf1 (R^178^NQK^181^) eliminates binding to PICK1 (Figure 2B). In contrast to wild-type (WT)-Arf1, this mutant (ΔCT-Arf1) has no effect on PICK1-Arp2/3 interactions (Figure 2C) or PICK1-actin interactions (Figure S2A). In order to utilize this mutant protein to investigate the role of the Arf1-PICK1 interaction in neurons, it is important to demonstrate that other properties of Arf1 apart from PICK1 binding are unaffected by deletion of the C-terminal four amino acids. Therefore, we compared the GTP-dependent binding of ΔCT-Arf1 and WT-Arf1 to a well-established Arf1 effector protein, Golgi-localized gamma-ear-containing Arf-binding protein 3 (GGA3; Myers and Casanova, 2008; Nie et al., 2003). ΔCT-Arf1 binds the VHS GAT domain of GGA3 in a GTP-dependent manner that is indistinguishable from that of WT-Arf1 (Figure 2D). We also compared the distribution of ΔCT-Arf1 and WT-Arf1 expressed in neurons, relative to each other and to a range of organelle marker proteins. Coexpression of ^myc^WT-Arf1 and ^HA^ΔCT-Arf1 demonstrates that the two proteins are identical in their subcellular localization in neuronal dendrites (Figure S2B). Expression of ^myc^WT-Arf1 or ^HA^ΔCT-Arf1 alone, followed by costaining for the recycling endosome marker Rab11, indicates that both WT- and ΔCT-Arf1 are partially localized to recycling endosomes (Figure S2C). WT- and ΔCT-Arf1 show similar partial colocalization with the postsynaptic density protein Homer, indicating that both WT- and ΔCT-Arf1 are localized to most, but not all, synapses (Figure S2D). Arf1 has an important function at the endoplasmic reticulum (ER)-Golgi interface (Dascher and Balch, 1994), so we analyzed colocalization with the Golgi resident protein giantin and the ER marker calreticulin in neuronal cell bodies. Both WT- and ΔCT-Arf1 show a similar partial overlapping distribution with calreticulin (Figure S2E) and weak colocalization with giantin (Figure S2F). Neither construct causes any detectable redistribution of ER or Golgi markers.

These experiments show that deletion of the extreme C-terminal four amino acids on Arf1 blocks its interaction with PICK1 but has no effect on its GTP-dependent binding to an alternative Arf1 effector protein or on its subcellular localization.

### Arf1 Regulates Actin Dynamics via PICK1 in Dendritic Spines

**Figure 3:**
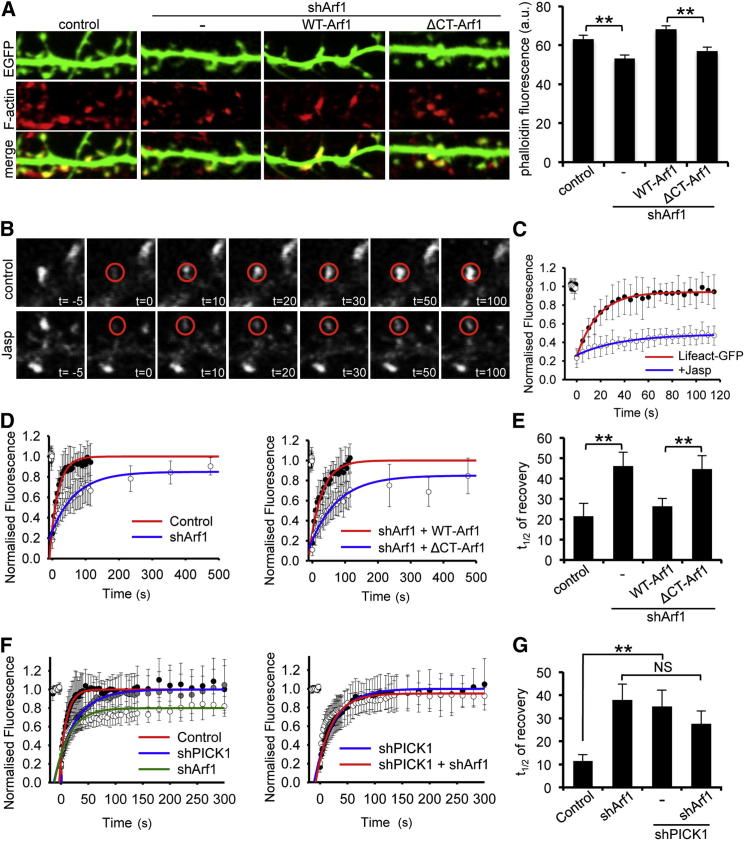
Arf1-PICK1 Interaction Regulates Actin Dynamics in Dendritic Spines (A) Arf1 knockdown and ΔCT-Arf1 expression results in reduced density of F-actin in dendritic spines. Dissociated hippocampal neurons transfected with plasmids expressing Arf1 shRNA and shRNA-resistant WT-Arf1 or ΔCT-Arf1 as well as EGFP were stained for F-actin using Alexa^647^-phalloidin. Image width is 15 μm. Spines of similar size and morphology were chosen for analysis. Graph shows fluorescence intensity of phalloidin staining in spine heads. n = 520–590 spines on 16–19 neurons from three independent experiments; values are mean ± SEM; ^∗∗^p < 0.001. (B) Fluorescence recovery after photobleaching (FRAP) of Lifeact-GFP-expressing spines is strongly inhibited by jasplakinolide (Jasp). Neurons were treated with 100 nM Jasp 20 min before bleaching. Representative image series are shown. The red circles represent the bleaching area and ROI for image analysis. Time units are seconds. (C) FRAP data for the experiment shown in (B). Data are fitted with single exponentials (colored lines). (D) Arf1 knockdown slows actin turnover measured by FRAP of Lifeact-GFP in dendritic spines (left). Coexpression of shRNA-resistant WT-Arf1 rescues the knockdown phenotype, whereas ΔCT-Arf1 does not rescue (right). Data are fitted with single exponentials (colored lines). (E) Averaged t_1/2_ for Arf1 knockdown and rescue conditions. n = 12–16; values are mean ± EM; ^∗∗^p < 0.01. (F) PICK1 knockdown blocks the effect of Arf knockdown on actin turnover. PICK1 shRNA alone causes a slowing of actin turnover (left). Under these conditions, the effect of Arf1 shRNA on the rate of actin turnover is blocked (right). Data are fitted with single exponentials (colored lines). Note the different timescale on the x axis, in order to better compare the curves on these graphs. (G) Averaged t_1/2_ for shArf1/shPICK1 combination experiments. n = 11–16; values are mean ± SEM; ^∗∗^p < 0.01. See also Figure S3.

Our data suggest that Arf1 regulates actin polymerization by modulating PICK1-mediated Arp2/3 inhibition. To test this hypothesis in neurons, we analyzed the levels of F-actin in dendritic spines using phalloidin conjugated to Alexa 647. Spines on neurons transfected with a previously characterized small hairpin RNA (shRNA) against Arf1 (Volpicelli-Daley et al., 2005) exhibit significantly reduced phalloidin staining compared to controls, which is rescued by coexpression of shRNA-resistant WT-Arf1 but not by ΔCT-Arf1 (Figure 3A). This suggests that Arf1 regulates F-actin levels via PICK1 in dendritic spines. F-actin undergoes a dynamic process of “treadmilling,” which involves the addition of actin monomers to the plus end of the filament and dissociation of monomers from the minus end. Recent studies have demonstrated that F-actin polymerization and depolymerization are highly regulated in dendritic spines (Hotulainen and Hoogenraad, 2010). To investigate this dynamic process, we used Lifeact-GFP, which binds F-actin in live cells, in conjunction with fluorescence recovery after photobleaching (FRAP) analysis. Expression of Lifeact-GFP in cultured hippocampal neurons results in a strong fluorescence signal in dendritic spine heads, consistent with the high levels of endogenous F-actin in spines (Figure S3B). FRAP of spine-localized Lifeact-GFP can be attributed to the formation of new F-actin and hence is a measure of endogenous actin turnover. To confirm that FRAP of Lifeact-GFP in spines is not the result of simple diffusion of fluorescent Lifeact-GFP through the spine neck and/or exchange with bleached Lifeact-GFP on existing actin filaments, we stabilized actin filaments using jasplakinolide and carried out FRAP analysis on Lifeact-GFP-expressing spines. Figures 3B and 3C show that under control conditions, fluorescence levels recover quite rapidly with t_1/2_ = 14.9 ± 2.4 s. Jasplakinolide application dramatically slows the recovery, resulting in t_1/2_ = 250 ± 31 s. The minimal recovery that persists under conditions in which actin filaments are stabilized is likely to represent a small amount of exchange of bleached Lifeact-GFP and fluorescent Lifeact-GFP on existing actin filaments. This important control experiment demonstrates that the vast majority of the FRAP recovery can be attributed to dynamic actin turnover in the spine. To investigate the role of Arf1 in actin dynamics, we carried out Lifeact-GFP FRAP analysis on dendritic spines expressing Arf1 shRNA. Spines of similar size and morphology were selected for all conditions. Arf1 knockdown results in a significantly slower recovery compared to controls (Figures 3D, 3E, and S3C), suggesting a role for Arf1 in regulating actin turnover in dendritic spines. Coexpression of shRNA-resistant WT-Arf1 rescues the knockdown phenotype to control levels, whereas shRNA-resistant ΔCT-Arf1 does not rescue (Figures 3D, 3E, and S3C), suggesting that Arf1-PICK1 interactions regulate actin turnover in dendritic spines.

To further support a role for Arf1 in regulating actin dynamics specifically via PICK1, we investigated the effect of Arf1 knockdown with PICK1 expression also knocked down. Lifeact-GFP FRAP analysis on dendritic spines expressing PICK1 shRNA indicates that PICK1 knockdown slows recovery, suggesting a reduction in the rate of actin turnover (Figures 3F, 3G, and S3D). Under conditions of reduced PICK1 expression, Arf1 knockdown has no effect on the rate of actin turnover (Figures 3F, 3G, and S3D). These results demonstrate that Arf1 regulates actin dynamics via PICK1 in dendritic spines.

### Arf1-PICK1 Interactions Regulate GluA2 Trafficking

**Figure 4:**
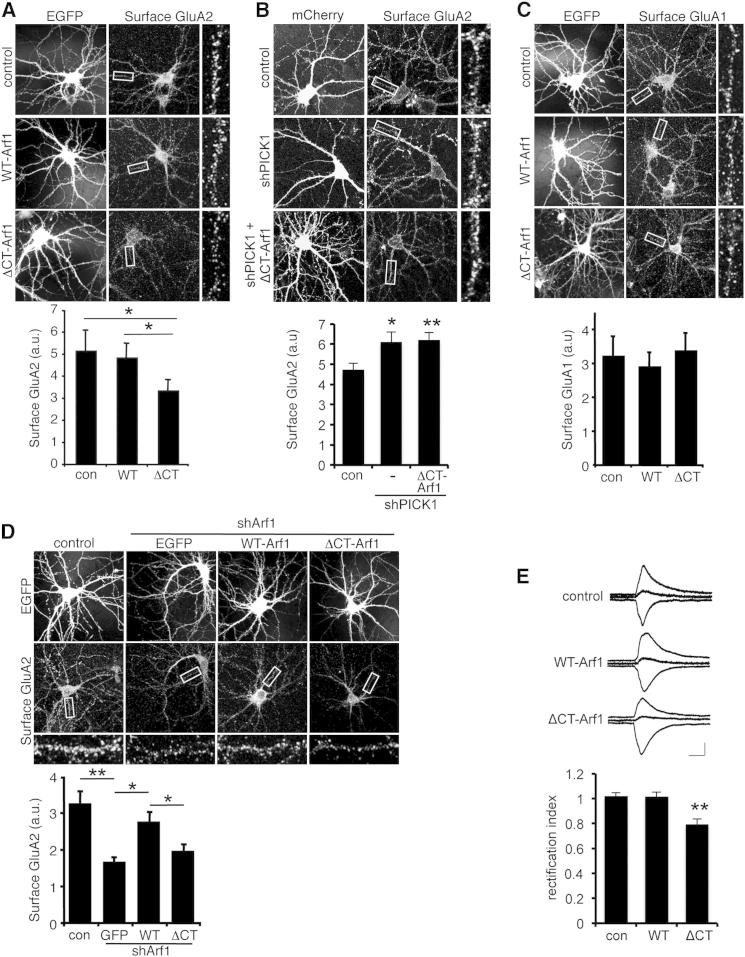
Arf1-PICK1 Interactions Are Required for Stable Surface Expression of GluA2-Containing AMPARs (A) ΔCT-Arf1 expression reduces GluA2 surface expression under basal conditions of synaptic activity. Dissociated hippocampal neurons transfected with either WT-Arf1-IRES-EGFP, ΔCT-Arf1-IRES-EGFP, or empty-IRES-EGFP were stained for surface GluA2. Graph shows fluorescence intensity of GluA2 surface staining. n = 18 cells from three independent experiments; values are mean ± SEM; ^∗^p < 0.02. (B) ΔCT-Arf1 expression has no effect on surface levels of GluA2 when PICK1 expression is knocked down by shRNA. Dissociated hippocampal neurons transfected with plasmids encoding mCherry alone or shPICK1 plus mCherry in conjunction with empty-IRES-EGFP or ΔCT-Arf1-IRES-EGFP were stained for surface GluA2. Graph shows fluorescence intensity of GluA2 surface staining. n = 16–21 cells from three independent experiments; values are mean ± SEM; ^∗^p < 0.05, ^∗∗^p < 0.01. (C) ΔCT-Arf1 expression has no effect on GluA1 surface expression. Dissociated hippocampal neurons transfected with either WT-Arf1-IRES-EGFP, ΔCT-Arf1-IRES-EGFP, or empty-IRES-EGFP were stained for surface GluA1. Graph shows fluorescence intensity of GluA1 surface staining. n = 15 cells from three independent experiments; values are mean ± SEM. (D) Arf1 knockdown reduces GluA2 surface expression, which is rescued by WT-Arf1 but not ΔCT-Arf1. Hippocampal neurons cotransfected with plasmids encoding Arf1 shRNA and either empty-IRES-EGFP, shRNA-resistant WT-Arf1-IRES-EGFP, or shRNA-resistant ΔCT-Arf1-IRES-EGFP were stained for surface GluA2. Graph shows fluorescence intensity of GluA2 surface staining. n = 17 cells from three independent experiments; values are mean ± SEM; ^∗^p < 0.02. (E) ΔCT-Arf1 expression causes synaptic expression of inwardly rectifying AMPARs. Bar graph shows mean ±SEM rectification index values for nontransfected cells (n = 23) and cells transfected with ΔCT-Arf1 (n = 15) or WT-Arf1 (n = 14). Insets show representative traces recorded at +40, 0, and −70 mV. Calibration bars for all traces shown are 50 pA/20 ms. Values are mean ± SEM; ^∗∗^p < 0.005. See also Figure S4.

Since PICK1-Arp2/3 interactions are involved in AMPAR trafficking (Rocca et al., 2008), we examined whether Arf1 can regulate this process via PICK1. To test this hypothesis, we analyzed the effect of removing the Arf1-dependent inhibitory drive on PICK1 by expressing the PICK1 nonbinding mutant ΔCT-Arf1 in hippocampal neurons and assayed surface levels of AMPAR subunit GluA2 by immunocytochemistry. While surface GluA2 in WT-Arf1-overexpressing cells is indistinguishable from controls, expression of ΔCT-Arf1 causes a marked reduction in surface GluA2 (Figure 4A). Total levels of GluA2 expression were unaffected by WT- or ΔCT-Arf1 expression (Figure S4A). To strengthen the conclusion that this is a PICK1-mediated effect, we exploited the observation that PICK1 requires synaptic activity to influence AMPAR trafficking and stimulate GluA2 internalization (Hanley and Henley, 2005; Nakamura et al., 2011; Terashima et al., 2008). Blockade of synaptic activity using TTX completely abolishes the ΔCT-Arf1-induced reduction in surface GluA2 (Figure S4B). The importance of the Arf1 C terminus and synaptic activity in these experiments strongly suggests that Arf1 inhibits PICK1-mediated trafficking of GluA2-containing AMPARs from the cell surface. To provide further support for this model, we investigated the effect of ΔCT-Arf1 under conditions of reduced PICK1 expression. PICK1 shRNA causes an increase in surface GluA2, as shown previously (Citri et al., 2010; Sossa et al., 2006), and completely blocks the effect of ΔCT-Arf1 expression (Figure 4B). This demonstrates that Arf1 regulates GluA2 surface expression via PICK1.

We explored the specificity of this effect and found that ΔCT-Arf1 does not affect surface expression of AMPAR subunit GluA1 (Figure 4C) or transferrin receptors (Figure S4C). These experiments show that the mechanism involving PICK1-Arf1 interactions is specific to the AMPAR subunit GluA2 and provide evidence that ΔCT-Arf1 expression has no effect on general trafficking events in neurons. Since Arf1 has important functions at the ER-Golgi interface (Dascher and Balch, 1994), we investigated the possibility that the observed effect of ΔCT-Arf1 on surface-expressed GluA2 could be a result of perturbations to trafficking at the ER. Importantly, neither WT-Arf1 nor ΔCT-Arf1 expression alters ER exit of GluA2 (Figure S4D), suggesting that forward traffic through the ER is unaffected by ΔCT-Arf1.

To further analyze the role of Arf1 in GluA2 trafficking, we knocked down endogenous Arf1 expression using shRNA. Arf1 knockdown leads to a dramatic decrease in surface levels of GluA2-containing AMPARs (Figure 4D), consistent with a role for Arf1 in blocking PICK1-mediated internalization of GluA2 under basal conditions. Neurons cotransfected with Arf1 shRNA and shRNA-resistant WT-Arf1 exhibit rescued levels of surface GluA2 comparable with the control. However, cotransfection with shRNA-resistant ΔCT-Arf1 does not rescue the shRNA-induced reduction in surface GluA2 (Figure 4D). We also used lentivirus to express Arf1 shRNA and shRNA-resistant Arf1 in neuronal cultures that were subjected to surface biotinylation to analyze GluA2 surface expression. The results are similar to the immunocytochemistry; Arf1 shRNA causes a reduction in surface GluA2, which is rescued by shRNA-resistant WT-Arf1 but not ΔCT-Arf1 (Figure S4E).

To assess the functional significance of the selective reduction in surface GluA2, we analyzed AMPAR-mediated synaptic transmission using whole-cell patch-clamp electrophysiological recordings in organotypic slices. We measured AMPAR excitatory postsynaptic currents (EPSCs) at three holding potentials (−70 mV, 0 mV, and +40 mV) and calculated the rectification index (RI) as the ratio of the slope 0 to +40 mV and −70 to 0 mV. Hence, RI < 1 corresponds to increased inward rectification. As expected, AMPAR EPSCs in nontransfected neurons show no detectable rectification, suggesting that most synaptic AMPARs contain GluA2 subunits. WT-Arf1 overexpression has no effect on RI, consistent with its lack of effect on GluA2 surface expression. In contrast, expression of ΔCT-Arf1 results in a significant inward rectification, indicative of the replacement of some GluA2-containing AMPARs with GluA2-lacking AMPARs at synapses (Figure 4E), demonstrating that Arf1-PICK1 interactions regulate synaptic GluA2 trafficking.

To assess the consequences of this alteration in AMPAR subunit composition for synaptic strength, we recorded EPSCs from transfected and nearby nontransfected neurons (in many cases simultaneously) in response to the same synaptic stimulus. Neither AMPAR nor NMDAR EPSC amplitude are affected by WT-Arf1 or ΔCT-Arf1 expression (Figures S4F and S4G), indicating that net synaptic strength is maintained constant following the replacement of some GluA2-containing AMPARs with GluA2-lacking AMPARs.

**Figure 5:**
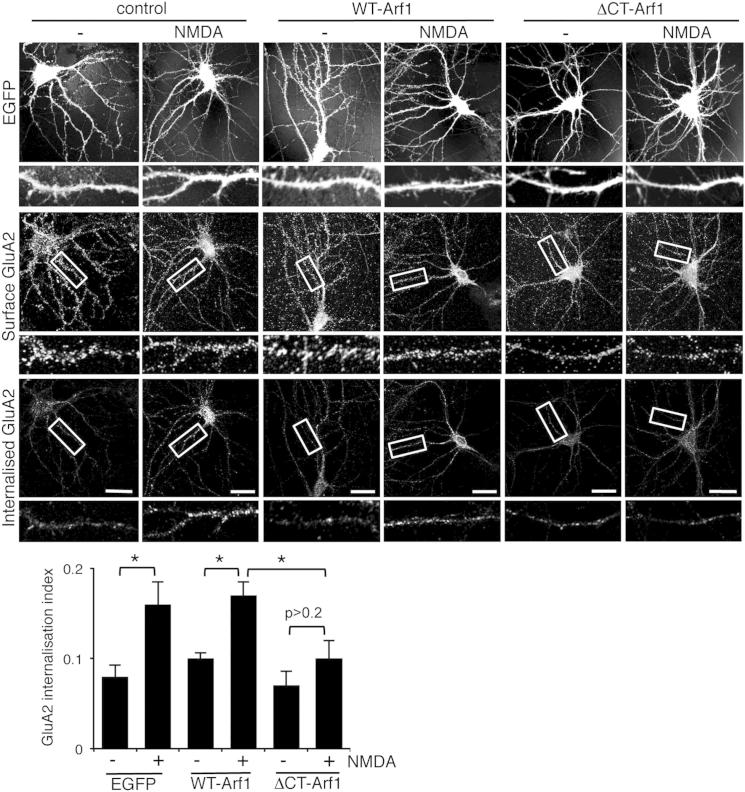
ΔCT-Arf1 Expression Occludes NMDA-Induced Internalization of GluA2-Containing AMPARs Dissociated hippocampal neurons were transfected with either WT-Arf1-IRES-EGFP, ΔCT-Arf1-IRES-EGFP or empty-IRES-EGFP plasmids. Internalized GluA2 in response to NMDA treatment (30 μm for 3 min; chemical LTD) in the absence of TTX was assayed using antibody feeding with anti-GluA2 antibodies. Cells were fixed and then stained for internal and surface pools of GluA2 using different fluorophore-conjugated secondary antibodies. Graph shows internalization of GluA2. n = 20 cells from three independent experiments; values are mean ± SEM; ^∗^p < 0.05.

Since the inhibition of Arp2/3 activity by PICK1 is a central mechanism of NMDA-stimulated AMPA receptor internalization (Rocca et al., 2008), we asked whether modulation of PICK1 by Arf1 is involved in this process. We used a “chemical LTD” protocol where NMDARs are activated by bath application of NMDA to promote AMPAR internalization, which is analyzed by antibody-feeding immunocytochemistry (Beattie et al., 2000). Control neurons show an approximately 2-fold increase in GluA2 internalization in response to NMDA treatment, which is unaffected by overexpression of WT-Arf1. However, in neurons expressing ΔCT-Arf1, NMDA-induced GluA2 internalization is abolished (Figure 5). A possible explanation for this result is that ΔCT-Arf1 interferes with the PICK1-GluA2 interaction. GluA2-PICK1 co-IPs are unaffected by the presence of ΔCT-Arf1, demonstrating that this is not the case (Figure S5).

Taken together, these data indicate that ΔCT-Arf1 expression causes GluA2 internalization under basal conditions, which occludes further AMPAR internalization in response to NMDA treatment. This suggests a model in which Arf1 limits PICK1-mediated internalization of surface GluA2-containing AMPAR and removal of this inhibitory drive is part of the mechanism involved in NMDA-induced AMPAR internalization.

### Arf1-PICK1 Interactions Regulate NMDAR-Dependent LTD

**Figure 6:**
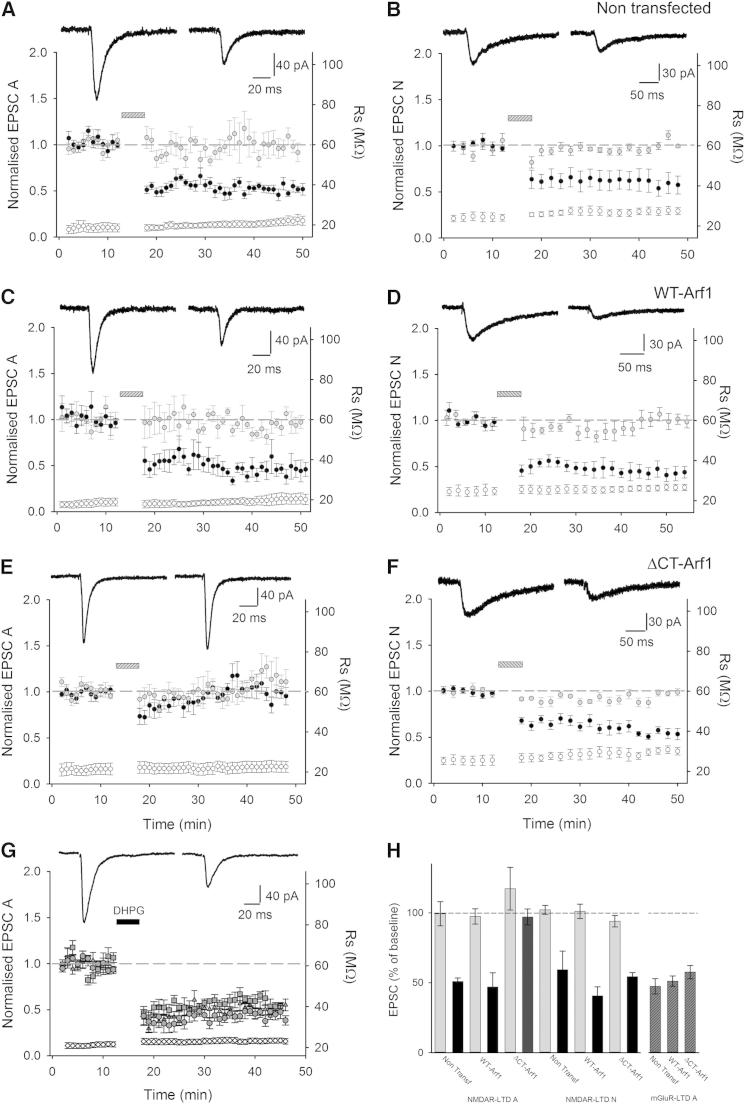
The Arf1-PICK1 Interaction Is Involved in NMDAR-Dependent LTD of AMPAR EPSCs (A) Stable AMPAR-mediated EPSCs (EPSC A) and input-specific NMDAR-dependent LTD was recorded in control (nontransfected) cells (n = 6). In (A)–(F), the left axis shows mean ± SEM values of EPSC amplitude normalized to baseline (closed circle, test input; gray circle, control input) and the right axis shows mean ± SEM series resistance values (open circle, Rser). The bar indicates delivery of a pairing protocol (1 Hz for 6 min, V_h_ = −40 mV). Insets show representative traces of the test input before and 25–30 min after LTD induction; each trace is an average of three consecutive EPSCs. (B) Pharmacologically isolated NMDAR EPSCs (EPSC N) also exhibited consistent, input-specific LTD when the same induction protocol was used (n = 5). (C) Input-specific LTD of EPSC A was consistently observed in cells transfected with WT-Arf1-IRES-EGFP (n = 6). (D) Input-specific LTD of EPSC N was consistently observed in cells transfected with WT-Arf1-IRES-EGFP (n = 5). (E) Input-specific LTD of EPSC A was completely abolished in cells transfected with ΔCT-Arf1-IRES-EGFP (n = 8). (F) Input-specific LTD of EPSC N was consistently observed in cells transfected with ΔCT-Arf1-IRES-EGFP (n = 4). (G) Bath application of S-DHPG in the presence of the NMDAR antagonist L-689,560 induced mGluR-dependent depression (mGluR-LTD) of similar amplitude in all experimental conditions. Left axis shows mean ± SEM values of EPSC amplitude normalized to baseline: (○) nontransfected n = 7, (Δ) WT-Arf1 n = 7, (□) ΔCT-Arf1 n = 5; right axis shows mean ± SEM series resistance values (⋄ Rser n = 19). Insets show representative traces from a cell transfected with ΔCT-Arf1-IRES-EGFP before and 25 min after DHPG application. (H) Bar graph summarizing the amplitude of LTD (plotted as percentage of baseline) for NMDAR-LTD (data from control input in gray and test input in black) and mGluR-LTD (patterned bars).

To more directly explore the role of the PICK1-Arf1 interaction in synaptic plasticity, we carried out electrophysiological recordings from CA1 pyramidal cells in organotypic slices, and a low-frequency stimulation pairing protocol was used to induce NMDAR-dependent LTD (Figure 6). Reliable LTD of AMPAR EPSCs can be induced in control nontransfected cells (Figure 6A) as well as in cells overexpressing WT-Arf1 (Figure 6C). In contrast, LTD is completely absent in ΔCT-Arf1-expressing neurons (Figure 6E), consistent with the AMPAR internalization assays shown in Figure 5. To investigate the specificity of this effect, we also tested NMDAR-dependent LTD of pharmacologically isolated NMDAR EPSCs. The same LTD protocol successfully induces a robust reduction in NMDAR EPSCs in control cells (Figure 6B), which is unaffected by WT-Arf1 expression (Figure 6D) and ΔCT-Arf1 expression (Figure 6F), providing additional evidence that ΔCT-Arf1 does not interfere with other neuronal trafficking or intracellular signaling pathways. As a further test for specificity, we investigated a form of mGluR-dependent LTD that is triggered by the application of dihydroxyphenylglycine (DHPG; Palmer et al., 1997). Application of the group 1 mGluR agonist DHPG results in a robust LTD of AMPAR EPSCs, which is unaffected by either WT-Arf1 or ΔCT-Arf1 expression (Figure 6G). This is consistent with a previous report suggesting that PICK1 is not involved in mGluR-LTD in the hippocampus (Citri et al., 2010). These experiments demonstrate that the interaction between Arf1 and PICK1 is specifically involved in NMDAR-dependent LTD of AMPAR EPSCs (Figure 6H).

### Arf1 Regulates Dendritic Spine Size via PICK1

**Figure 7:**
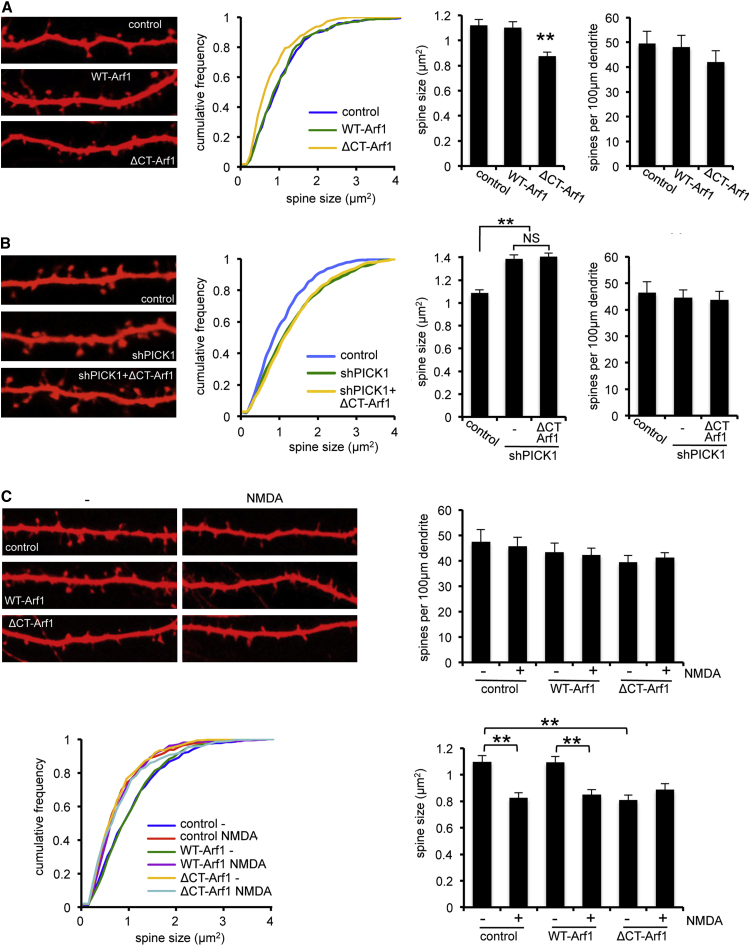
The Arf1-PICK1 Interaction Regulates Dendritic Spine Size (A) ΔCT-Arf1 expression decreases basal spine size. Dissociated hippocampal neurons were transfected with plasmid encoding mCherry as a morphological marker as well as either WT-Arf1, ΔCT-Arf1, or empty vector. Image width is 30 μm. Graphs show quantification of linear spine densities and spine size. Histograms show mean ± SEM. Spine size for the ΔCT-Arf1 condition is significantly reduced compared to control and WT-Arf1 conditions (p < 0.001, Kolmogorov-Smirnov [K-S] test). (B) PICK1 knockdown blocks ΔCT-Arf1-mediated spine shrinkage. Dissociated hippocampal neurons were transfected with plasmids encoding mCherry or shPICK1 plus mCherry as well as either ΔCT-Arf1 or empty vector. Image width is 30 μm. Graphs show quantification of linear spine densities and spine size. Histograms show mean ± SEM. ΔCT-Arf1 has no effect on spine size when PICK1 expression is knocked down by shRNA. (C) ΔCT-Arf1 expression mimics and occludes NMDA-induced spine shrinkage. Hippocampal neurons were transfected as in (A) and subjected to a chemical LTD treatment. Image width is 30 μm. Graphs show quantification of linear spine densities and spine size. Histograms show mean ± SEM. NMDA application causes a significant reduction in spine size in control and WT-Arf1 conditions (p < 0.001, K-S test). NMDA has no effect on spine size in neurons expressing ΔCT-Arf1 (p > 0.1, K-S test).

Since PICK1 restricts spine size via inhibition of the Arp2/3 complex (Nakamura et al., 2011), we investigated whether Arf1 can modulate dendritic spine size via PICK1. While dendritic spines in WT-Arf1-overexpressing cells are indistinguishable from controls, expression of ΔCT-Arf1 causes a marked reduction in the size of spines (Figure 7A). This strongly suggests that Arf1 binding to PICK1 modulates dendritic spine size under basal conditions. Expression of neither protein affects the density of spines on dendrites (Figure 7A). To provide further evidence that Arf1 functions via PICK1 to regulate spine size, we examined the effect of ΔCT-Arf1 under conditions of reduced PICK1 expression. Neurons transfected with PICK1 shRNA have significantly larger spines compared to controls, as shown previously (Bassani et al., 2012; Nakamura et al., 2011). Importantly, ΔCT-Arf1 has no effect on spine size in neurons expressing PICK1 shRNA (Figure 7B), demonstrating that the regulation of spine size by Arf1 requires PICK1. As well as regulating basal spine size, PICK1 is required for spine shrinkage during chemical LTD (Nakamura et al., 2011); therefore, we examined the effect of Arf1 on this process. As shown in Figure 7A, ΔCT-Arf1 causes a reduction in spine size, which is similar to the shrinkage observed in response to NMDAR activation during chemical LTD (Figure 7C). We therefore investigated whether these treatments occlude each other. In agreement with this hypothesis, NMDAR activation has no effect on spine size in neurons expressing ΔCT-Arf1 (Figure 7C), suggesting that NMDA-induced spine shrinkage involves the Arf1-PICK1 pathway. In contrast, NMDA-induced spine shrinkage is unaffected by WT-Arf1 overexpression. NMDAR activation does not affect the density of spines on dendrites within the time period tested here, as shown previously (Figure 7C; Nakamura et al., 2011). These results demonstrate a crucial role for Arf1-PICK1 interactions in maintaining dendritic spine size and suggest that Arf1 restricts spine shrinkage via interaction with PICK1.

### Arf1 Activation Is Regulated by NMDAR Stimulation

**Figure 8:**
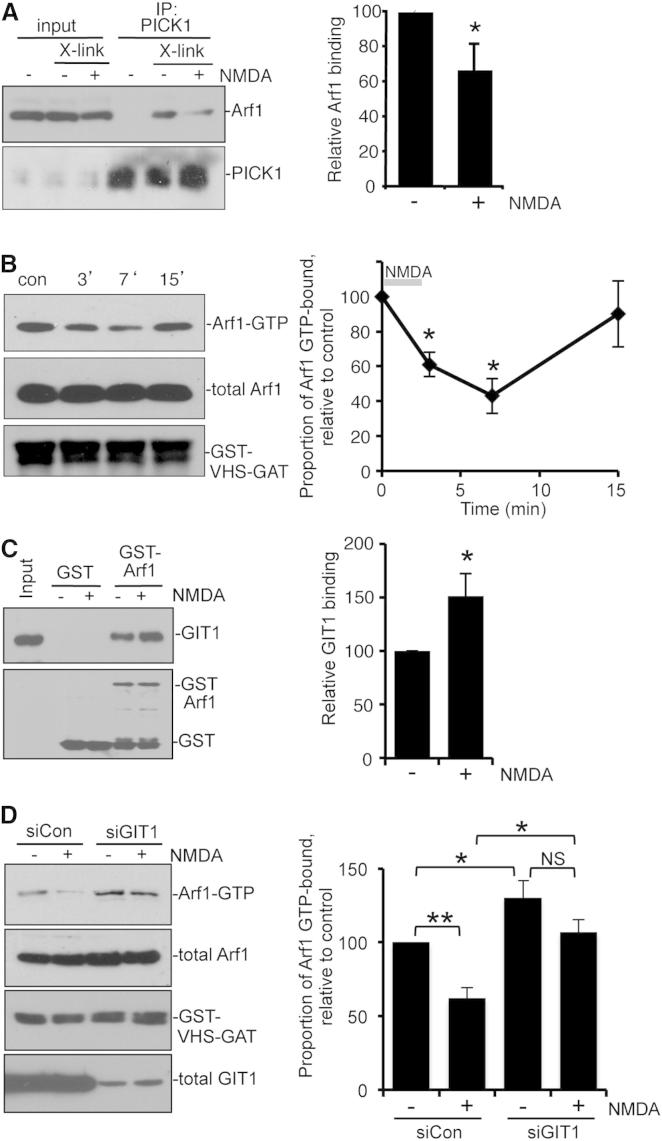
Arf1 Activation and Binding to PICK1 Are Regulated by NMDAR Activation via the Arf GAP GIT1 (A) NMDAR stimulation inhibits the PICK1-Arf1 interaction in neurons. Cultured neurons were exposed to NMDA for 3 min then returned to normal medium for a further 7 min. Cultures were fixed in paraformaldehyde (see Experimental Procedures), quenched, and lysed. Extracts were subjected to immunoprecipitation with anti-PICK1 antibodies and bound proteins detected by western blotting using specific antibodies as shown. Graph shows quantification of Arf1 bound to PICK1. n = 6; values are mean ± SEM; ^∗^p < 0.05. (B) NMDAR stimulation transiently decreases levels of activated Arf1 in neurons. Cultured neurons were exposed to NMDA for 3 min and then returned to normal medium for the indicated times. Cells were lysed and extracts incubated with immobilized GST-VHS-GAT. Total and bound proteins were detected by western blotting using anti-Arf1 and anti-GST. Graph shows the fraction of Arf1 that is GTP bound, relative to control. n = 5; values are mean ± SEM; ^∗^p < 0.02. (C) NMDAR stimulation increases GIT1 binding to Arf1. Cultured neurons were exposed to NMDA for 3 min, lysed, and extracts incubated with immobilized GST-Arf1. Bound proteins were detected by western blotting using anti-GIT1 and anti-GST. Graph shows quantification of GIT1 bound to GST-Arf1, relative to control (no NMDA). n = 6; values are mean ± SEM; ^∗^p < 0.05. (D) NMDA-induced deactivation of Arf1 requires GIT1. Cultured neurons were transfected with siRNA against GIT1 or a control siRNA. Then 6–8 days later, cultures were exposed to NMDA for 3 min and then returned to normal medium for 7 min. Cells were then lysed and extracts incubated with immobilized GST-VHS-GAT. Total and bound proteins were detected by western blotting using anti-Arf1, anti-GIT1, and anti-GST. Graph shows the fraction of Arf1 that is GTP bound relative to control. n = 6; values are mean ± SEM; ^∗^p < 0.05, ^∗∗^p < 0.01.

Since LTD expression involves AMPAR internalization and spine shrinkage, both of which are inhibited by Arf1 under basal conditions, this blockade by Arf1 must be removed during LTD induction. To test this, we investigated whether NMDAR stimulation affects the PICK1-Arf1 interaction by carrying out co-IPs from cultured neuronal extracts following chemical LTD. A crosslinking protocol (see Experimental Procedures) was utilized to preserve native complexes, which would otherwise dissociate after lysis in the absence of GTPγS. Activating NMDARs leads to a significant decrease in the PICK1-Arf1 interaction compared to untreated cells (Figure 8A). Since Arf1 binds PICK1 in a GTP-dependent manner, we asked whether the reduction in Arf1 binding was due to a decreased proportion of activated (GTP-bound) Arf1 following NMDAR stimulation. Pull-down assays were performed using the VHS-GAT domain of GGA3 to monitor levels of activated Arf1 in extracts from NMDA-treated cultured neurons. Following bath application of NMDA, there is a transient decrease of around 60% in levels of activated Arf1 at 7 min after the initial NMDA application (Figure 8B). These experiments demonstrate that NMDAR activation inhibits PICK1-Arf1 interactions by reducing Arf1-GTP levels on a timescale that is consistent with that of AMPAR internalization during chemical LTD (Ashby et al., 2004). The activation state of small GTPases is regulated by GEFs, which exchange GDP for GTP, hence switching the protein “on,” and GAPs, which stimulate the catalysis of GTP to GDP, switching the protein “off.” Our data suggest that a GAP may be recruited to deactivate Arf1 in response to NMDA treatment. GIT1 is an Arf GAP that has been shown to play a role in both AMPAR trafficking and dendritic spine morphogenesis (Ko et al., 2003; Zhang et al., 2003). Therefore, we investigated whether GIT1 regulates Arf1 activation during chemical LTD. We used GST-Arf1 pull-downs to investigate Arf1-GIT1 binding in response to NMDAR stimulation. Figure 8C shows that GIT1 binding to GST-Arf1 increases significantly following NMDA application, suggesting that GIT1 regulates Arf1 in response to NMDAR stimulation. To directly test the role of GIT1 in NMDA-induced Arf1 deactivation, we used small interfering RNA (siRNA) to knock down GIT1 expression in cultured neurons and analyzed GTP-Arf1 levels by pull-down assays using the VHS-GAT domain of GGA3. GIT1 knockdown blocks the NMDA-induced reduction in Arf1-GTP levels (Figure 8D). In addition, GIT1 knockdown causes an increase in GTP-Arf1 under basal conditions, indicating that GIT1 is tonically active in neurons to regulate Arf1 activation (Figure 8D).

These results demonstrate that GIT1 is critical for Arf1 deactivation during chemical LTD.

## Discussion

Here, we describe a mechanism by which Arf1 regulates actin dynamics and membrane trafficking via an interaction with PICK1. We show that activated Arf1 directly binds PICK1 to block the inhibition of Arp2/3-dependent actin polymerization. Under basal conditions of synaptic activity, GTP-bound Arf1 suppresses PICK1-mediated inhibition of Arp2/3 activity, limiting spine shrinkage and AMPAR internalization. Following NMDAR stimulation, Arf1 is deactivated by the ArfGAP GIT1, allowing PICK1 to inhibit Arp2/3 activity and consequently promote AMPAR internalization and contribute to spine shrinkage, which are crucial aspects of LTD expression (Figure S6).

Disruption of this pathway by Arf1 knockdown or expression of the PICK1 nonbinding mutant of Arf1 leads to a slowing of actin turnover in dendritic spines, spine shrinkage, and internalization of surface-expressed GluA2-containing AMPARs. The reduction in surface GluA2 levels and spine size following the loss of Arf1-dependent inhibitory drive on PICK1 occludes subsequent NMDAR-dependent AMPAR internalization and spine shrinkage.

### Arf1-Regulated AMPAR Trafficking and Synaptic Plasticity

Our data show that the expression of ΔCT-Arf1 causes a PICK1-dependent loss of surface GluA2 and consequent expression of inwardly rectifying synaptic AMPARs by removing the Arf1-dependent inhibitory drive on PICK1. LTD involves the internalization of a pool of GluA2 that is regulated by PICK1 (Hanley and Henley, 2005; Terashima et al., 2008). Therefore, our observations can be explained by a model in which ΔCT-Arf1 expression causes GluA2 trafficking events that occlude subsequent NMDAR-mediated internalization of GluA2-containing AMPARs during LTD. The pool of GluA2-containing AMPARs internalized as a result of ΔCT-Arf1 expression is presumably the same pool of GluA2 that would be internalized during LTD, given their mutual dependence on endogenous PICK1. However, the lack of effect of ΔCT-Arf1 on AMPAR-EPSC amplitude indicates that there is a compensatory mechanism that keeps synaptic strength constant. The observed rectification change suggests that this is due to the replacement of GluA2-containing AMPARs with GluA2-lacking AMPARs. Consistent with this hypothesis, PICK1 overexpression also causes a reduction in surface GluA2 and inward rectification (Nakamura et al., 2011; Terashima et al., 2004). This is associated with an increase in AMPAR-EPSC amplitude because of the insertion of a large number of high-conductance GluA2-lacking AMPARs. As expected, the effect of PICK1 overexpression is greater than that of ΔCT-Arf1, which increases the activity of endogenous PICK1. The difference in PICK1 activity under these two sets of conditions can explain the differences in the level of rectification and also the extent to which the AMPAR-EPSC amplitude is altered. For ΔCT-Arf1, our observations are most compatible with a mechanism in which the internalization of GluA2-containing AMPAR is balanced by the incorporation of a smaller number of higher-conductance GluA2-lacking AMPARs. Therefore, we conclude that there is an occlusion of part of the LTD machinery, specifically activation of PICK1, to inhibit the Arp2/3 complex and hence drive GluA2 internalization.

We see no effect of WT-Arf1 overexpression on actin dynamics, AMPAR trafficking, LTD, or spine morphology. A likely explanation for this is that absolute levels of Arf1 are not a limiting factor, but instead the activities of upstream regulators (e.g., the ArfGAP GIT1) are the major influence. Therefore, increasing the absolute levels of WT-Arf1 by overexpression has no effect without modulation of GAP or GEF activity.

### Arf1 Regulates Actin Polymerization via Its Effector Protein PICK1

In dendritic spines, Arf1 knockdown or ΔCT-Arf1 expression leads to reduced density of actin filaments and slower F-actin turnover. The most straightforward explanation for this result is that removing the inhibitory influence of Arf1 on PICK1 permits PICK1-mediated inhibition of Arp2/3-mediated actin polymerization. Since PICK1 inhibits Arp2/3 activity, PICK1 knockdown might be expected to increase the rate of actin turnover as a result of increased Arp2/3 activity. However, we show that PICK1 knockdown slows actin turnover. This is similar to the effect of cofilin knockdown (also known as actin depolymerizing factor, or ADF) reported previously (Hotulainen et al., 2009). Cofilin causes depolymerization of actin filaments, yet cofilin knockdown leads to a slowing of actin turnover in dendritic spines. This can be explained by a depleted pool of available G-actin when actin dynamics are shifted in favor of F-actin, which would occur under conditions of reduced PICK1 or cofilin expression. Importantly, the effect of Arf1 knockdown on actin dynamics is blocked by PICK1 shRNA, indicating that this effect of Arf1 is mediated by PICK1.

Our in vitro data show that Arf1 blocks inhibition of Arp2/3 activity by PICK1. We propose that the mechanism behind this blockade involves an Arf1-induced conformational change in PICK1 to enhance the PDZ-BAR domain intramolecular interaction leading to a “closed” conformation of PICK1, which reduces its subsequent binding to and inhibition of the actin-nucleation machinery (Rocca et al., 2008). Arf1 binds the PDZ domain of PICK1; however, Arf1 does not compete with GluA2 for PICK1 interactions. This suggests that Arf1 does not bind to the canonical PDZ domain carboxylate loop but has a distinct binding site within the PDZ domain. This hypothesis is supported by the Arf1 C-terminal sequence, which does not conform to any known consensus PDZ domain binding motif (Harris and Lim, 2001).

It is becoming increasingly apparent that Arf proteins have important functions in the organization of the actin cytoskeleton (Myers and Casanova, 2008). Previous reports have focused on the indirect effects of Arf1 on signaling pathways controlled by small Rho-family GTPases. For example, Arf1-dependent recruitment of COPI at the Golgi leads to Arp2/3-dependent actin polymerization via a pathway involving the Arf1-activated Cdc42 GAP ARHGAP10 and consequent recruitment of the Cdc42 effector N-WASP (Dubois et al., 2005). In contrast, in the mechanism described here, Arf1 modulates the activity of the Arp2/3 complex by direct binding to the Arp2/3 inhibitor PICK1, defining PICK1 as an Arf1 effector. Therefore, this mechanism does not rely on Rho GTPase signaling pathways and represents an alternative pathway for regulating actin polymerization. Since GTP-bound Arf1 blocks the inhibition of Arp2/3 activity by PICK1, our model is consistent with the hypothesis that activated Arf1 is a positive regulator of actin polymerization (Dubois et al., 2005; Heuvingh et al., 2007; Myers and Casanova, 2008).

In conclusion, this study identifies an important role for Arf1 in neurons distinct from its well-established role as a regulator of vesicle trafficking in the Golgi. Arf1 signaling regulates Arp2/3-mediated actin polymerization via an effector protein, PICK1, to control AMPAR trafficking and dendritic spine size. Furthermore, it defines an important signaling pathway whereby NMDAR activation leads to activation of GIT1, which inhibits Arf1 and thereby activates PICK1 to inhibit Arp2/3-mediated actin polymerization, a process that is required for AMPAR internalization during LTD. Since the dynamic actin cytoskeleton is essential to the control of a number of processes in cell biology, it is possible that the GIT1-Arf1-PICK1-Arp2/3 pathway may be a pivotal mechanism for regulating actin polymerization in other processes related to neuronal function.

## Experimental Procedures

### Coimmunoprecipitation

Co-IPs were performed as previously described (Rocca et al., 2008; Nakamura et al., 2011). Extracts of cortical neuronal cultures, HEK293, or COS7 cells were prepared in lysis buffer and subsequently incubated with either 2 μg anti-PICK1, anti-FLAG, anti-GluA2, or control immunoglobulin G (IgG) antibodies. For competition assays, EGFP- and pep2-EVKI were expressed in neurons using Sindbis virus. For crosslinking experiments, cultured neurons were treated with 50 μM NMDA and chased for the indicated times followed by fixation in 1% paraformaldehyde. After quenching with glycine, neurons were prepared in lysis buffer for subsequent immunoprecipitation using anti-PICK1 antibodies and processed for western blotting. Paraformaldehyde crosslinking has been shown not only to promote stabilization of transient protein-protein interactions in close proximity to each other but also to allow stringent conditions during cell lysis to minimize false positives. Moreover, formaldehyde crosslinks are reversible during sample preparation for SDS-PAGE by boiling in Laemmli buffer (Klockenbusch and Kast, 2010).

### Preparation of Recombinant Proteins

His_6_ and GST fusions were expressed and purified essentially as described previously in Rocca et al. (2008).

### GST Pull-Down Assays

Pull-down assays were conducted as described in Rocca et al. (2008).

### Actin Polymerization Assays

Polymerization reactions were carried out essentially as described in Rocca et al. (2008).

### Primary Neuronal Cultures

All experiments were performed in accordance with Home Office guidelines as directed by the Home Office Licensing Team at the University of Bristol. Rat embryonic hippocampal neuronal cultures were prepared from E18 Wistar rats using standard procedures. The culture medium was Neurobasal medium (Gibco) supplemented with B27 (Gibco) and 2 mM glutamine. Neurons were transfected with plasmid DNA at days in vitro (DIV) 11–13 (unless otherwise stated) using Lipofectamine 2000 (Invitrogen) and used for experiments 4–6 days later or with siRNA at DIV 7–8 using RNAiMAX (Invitrogen) and used for experiments 6–8 days later.

### Immunocytochemistry

For surface staining of AMPARs, neurons were treated with or without 1 μM TTX for 1 hr, fixed in 4% paraformaldehyde plus 4% sucrose (PFA) for 5 min, and then labeled with anti-AMPAR subunit antibodies followed by staining with mouse-anti Cy3 secondaries. For antibody feeding experiments, live hippocampal neurons (DIV 15–20) were surface labeled with anti-GluA2 (Millipore) antibodies for 30 min at room temperature in HBS in the absence of TTX. Neurons were then washed in HBS and treated with 50 μM NMDA for 3 min at 37°C followed by a 10 min chase without drugs. Neurons were fixed for 5 min with PFA and stained with anti-mouse Cy5 secondaries. After a 20 min fixation in PFA, cells were permeabilized and stained with anti-mouse Cy3 secondaries. Images were acquired on a LSM510 confocal microscope (Zeiss) and analyzed using NIH Image J. Internalization index was calculated by dividing the value corresponding to internalized staining by the value corresponding to total staining (internalized + surface). The GFP signal was used as a mask, and the average fluorescence intensity was measured within this area. For whole-cell staining, neurons were fixed for 20 min in PFA, permeabilized, and incubated with antibodies or with phalloidin conjugated to Alexa 647.

### Fluorescence Recovery after Photobleaching

Neurons transfected with Lifeact-GFP constructs as specified were imaged at 37°C in HBS buffer using a Zeiss LSM510 confocal microscope (Figures 3B–3E and S3C) or a Perkin Elmer Ultraview spinning disc microscope (Figures 3F, 3G, and S3D). Image conditions were optimized to minimize photobleaching induced by time-lapse imaging. Bleaching was achieved at maximum laser transmission at 488 nm for less than 30 s and targeted to predefined circular regions of interest (ROIs) of approximately 3 μm radius corresponding to individual spines of similar size and morphology. Following bleaching, images were automatically acquired at 5 s intervals, unless otherwise stated. Background fluorescence was subtracted for each frame during the image processing to quantify the recovery. Recovery at time point t was calculated as ROI/REF, where ROI is intensity at the region of interest and REF corresponds to intensity of a “reference” nearby spine to account for minor focus changes during acquisition. Recovery values were normalized to the average intensity of five prebleach frames. Exponential fit to simple regression curves was performed with Sigmaplot software. Values were fit to the equation y = y_0_ + a(1 − exp^(−bx)^), where y_0_, a, and b are offset, maximum value, and time constant, respectively. To optimize the fit, all curves analyzed were constrained to reach the maximum value of recovery (a + y0), defined as the average of the last three values. The equation t_1/2_ = ln(0.5)/−b was used to extract half-life of recovery, and conditions were compared using a t test.

### Electrophysiology

Organotypic slices were prepared from P8 Wistar rats using the interface method (Bortolotto et al., 2011; Stoppini et al., 1991). Transverse hippocampal slices (400 μm) were placed on Millicell culture plate inserts (Millipore) and maintained at 35°C, 5% CO_2_ in MEM-based culture media containing 20% horse serum and (in mM): 30 HEPES, 16.25 glucose, 5 NaHCO_3_, 1 CaCl_2_, 2 MgSO_4_, 0.68 ascorbic acid, and 1 μg/ml insulin (pH 7.28), 320 mOsm. Biolistic transfection was performed using a Helios GeneGun (Bio-Rad) and electrophysiological recordings were performed, blind with respect to the transfected plasmid (either WT-Arf1-IRES-EGFP or ΔCT-Arf1-IRES-EGFP together with mCherry), 2–4 days later. Whole-cell voltage-clamp recordings were made from CA1 pyramidal cells (Vh = −70 mV) at 6–11 DIV. Patch pipettes contained (in mM) 115 Cs-methanesulfonate, 20 CsCl, 10 HEPES, 2.5 MgCl_2_, 4 Na_2_ATP, 0.4 Na_3_GTP, 10 sodium phosphocreatine, and 0.6 EGTA or alternatively 8 NaCl, 130 Cs-methanesulfonate, 10 HEPES, 0.5 EGTA, 4 MgATP, 0.3 Na_3_GTP, and 5 QX-314 (pH 7.25, 290 mOsm). Picrotoxin (50–100 μM) and 2-chloroadenosine (1–2 μM) were routinely included in the bath solution (124 mM NaCl, 3 mM KCl, 26 mM NaHCO_3_, 1.4 mM NaH_2_PO_4_, 4 mM CaCl_2_, 4 mM MgSO_4_, 10 mM glucose; saturated with 95% O_2_/5% CO_2_). Bath temperature was maintained at 25°C–28°C. In order to isolate NMDA EPSCs, 3 μM NBQX was added and Vh = −40 mV; in some cases, D-AP5 (50 μM) was added to confirm that synaptic responses were NMDAR mediated. When measuring RI, 100 μM spermine was added to the intracellular solution in order to prevent dilution of cytoplasmic polyamines and 50–100 μM AP5 was added to the bath solution. RI was calculated as the ratio of the slope 0–40 mV and −70 to 0 mV; the average EPSC (−70 mV) was averaged with the one following the depolarization period. Two stimulating electrodes were placed in the Schaffer collateral-commissural pathway and stimulated at 0.05–0.1 Hz to record AMPAR EPSCs and at 0.03 Hz for NMDAR EPSCs. When investigating mGluR-LTD, L-689,560 (5 μM) was added to the bath solution and (S)-3,5-DHPG (100 μM) was bath applied for 5 min. Data were acquired and analyzed with WinLTP (Anderson and Collingridge, 2007). Average amplitudes of EPSCs over a period of 5 min immediately before and 25 min after LTD were considered to determine the magnitude of LTD. Statistical analysis was performed using the Student’s t test or one-way ANOVA as appropriate, and significance was set at p < 0.05.

See Supplemental Experimental Procedures for further details.

## Supplemental Information

Document S1. Figures S1–S6 and Supplemental Experimental Procedures
