# Supplementary material for: The Small GTPase Arf1 Modulates Arp2/3-Mediated Actin Polymerization via PICK1 to Regulate Synaptic Plasticity
Source: Neuron. 2013 Jul 24;79(2):293–307. doi: 10.1016/j.neuron.2013.05.003 (PMC3725416; doi:10.1016/j.neuron.2013.05.003)
Supplement: Document S1. Figures S1–S6 and Supplemental Experimental Procedures [file mmc1.pdf]

Neuron, Volume *79*

Supplemental Information

The Small GTPase Arf1 Modulates Arp2/3-Mediated Actin Polymerization via PICK1 to Regulate Synaptic Plasticity

Daniel L. Rocca, Mascia Amici, Anna Antoniou, Elena Blanco Suarez, Nagaraj Halemani, Kai Murk, Jennifer McGarvey, Nadia Jaafari, Jack R. Mellor, Graham L. Collingridge, and Jonathan G. Hanley

SUPPLEMENTAL FIGURES

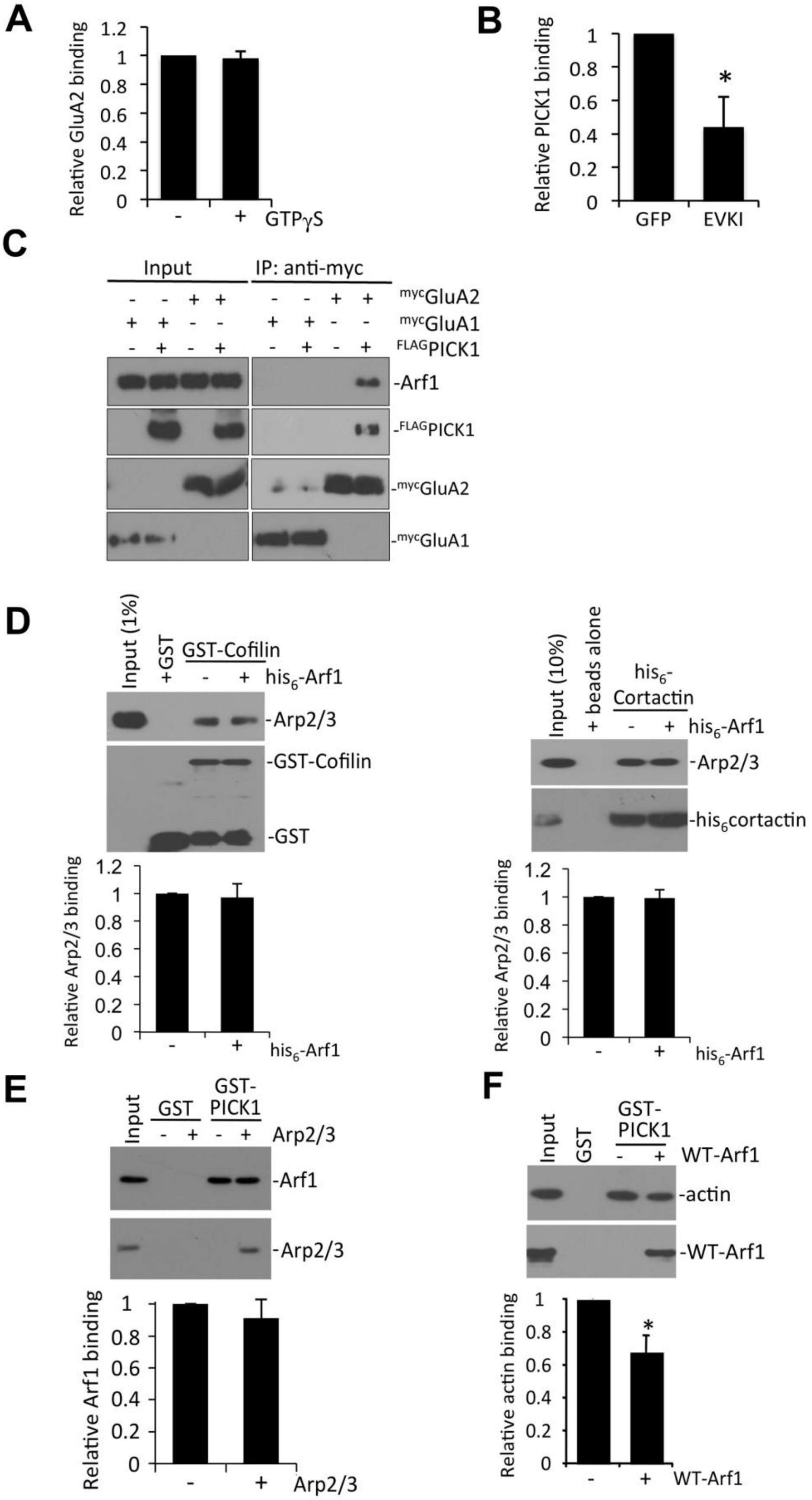

Figure S1 (Related to Figure 1)

A) Endogenous PICK1-GluA2 binding is unaffected by the presence of GTPγS. Graph shows quantification of the GluA2 bands for the PICK1 co-immunoprecipitation presented in Figure 1C (n=4). Values are mean ± SEM.

B) Pep2-EVKI inhibits GluA2-PICK1 interaction. Graph shows quantification of the PICK1 bands for the co- immunoprecipitation presented in Figure 1D (n=5), $p<0.05$. Values are mean ± SEM.

C) Endogenous Arf1 co-immunoprecipitates with PICK1 and GluA2, but not GluA1 in HEK293 cells. Cells were transfected with plasmids encoding $PICK1^{flag}$ and $^{myc}GluA1$ or $^{myc}GluA2$. 1% Triton X-100 lysates were immunoprecipitated with anti-myc antbody, and bound proteins were detected by western blotting.

D) Activated Arf1 has no effect on Arp2/3 binding to cofilin (left) or cortactin (right). Immobilised GST-Cofilin or his_6cortactin were complexed with 10 nM purified Arp2/3 complex in the absence or presence of GTPγS-bound his_6Arf1. After washing, bound proteins were detected by immunoblotting. Top panels show representative western blots, graphs show quantification of Arp2/3 binding to GST-cofilin or his_6cortactin (n=5). Values are mean ± SEM.

E) The Arp2/3 complex does not inhibit the interaction of Arf1 with PICK1. Immobilised GST-PICK1 was incubated with GTPγS-bound his_6Arf1 in the absence or presence of 50 nM purified Arp2/3 complex. Top panels show representative western blots, graphs show quantification of Arf1 binding to GST-PICK1 (n=5). Values are mean ± SEM.

F) Arf1 inhibits the PICK1-actin interaction. Immobilised GST-PICK1 in the absence or presence of GTPγS-bound his_6-Arf1 was incubated with 200 μg of brain homegenate. Bound proteins were detected by immuoblotting. Graphs show quantification of actin bound to GST-PICK1 (n=4), $*p<0.01$. Values are mean ± SEM.

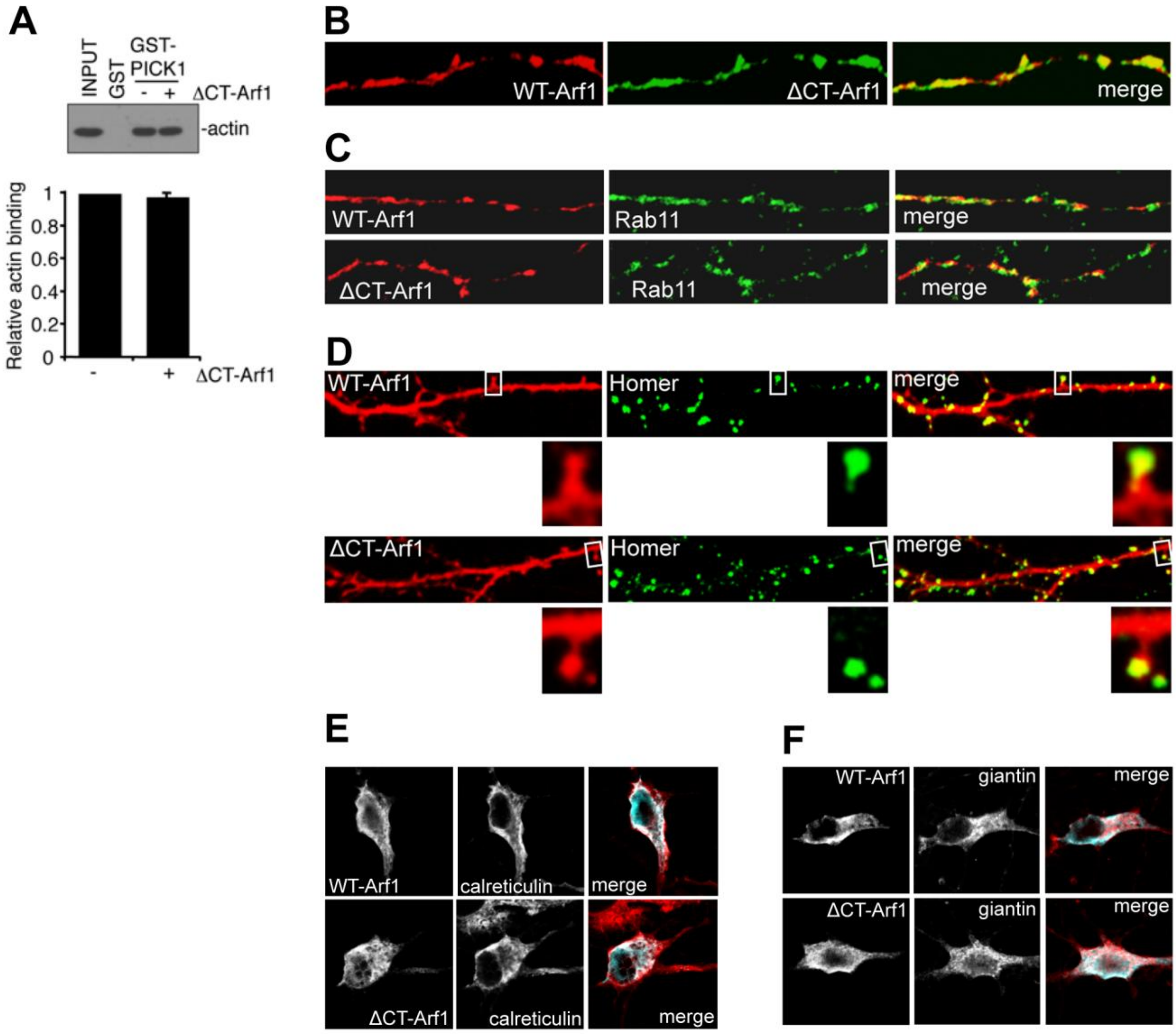

Figure S2 (Related to Figure 2)

(A) ΔCT-Arf1 does not inhibit the PICK1-actin interaction. Immobilised GST-PICK1 in the absence or presence of GTPγS-bound ΔCT-Arf1 was incubated with 200 μg brain homegenate. Bound proteins were detected by immunoblotting. Graphs show quantification of actin bound to GST-PICK1 (n=4). Values are mean ± SEM.

(B) The ΔCT mutation has no effect on the subcellular localization of Arf1. Neurons transfected with ^{myc}WT-Arf1 and ^{HA}ΔCT-Arf1 were stained with anti-myc (rabbit, red channel) and anti-HA (mouse, green channel) antibodies. A representative section of dendrite is shown (image width 30 μm).

(C-F) The ΔCT mutation has no effect on the colocalization of Arf1 with a range of organelle markers. Neurons were transfected with either ^{myc}WT-Arf1 or ^{HA}ΔCT-Arf1 and stained for the epitope tag as well as

an endogenous protein as specified. Dendrite segments are shown for F and G (image width 40 μm), cell bodies for H and I (image width 60 μm).

(C) Rab11 (recycling endosome).

(D) Homer (postsynaptic density). Note that a higher gain setting is needed to detect Arf1 proteins in dendritic spines, hence the defined structures in the dendritic shaft are not discernible. Bottom panels show zoomed image of single spines (image width 4μm).

(E) Giantin (Golgi)

(F) Calreticulin (endoplasmic reticulum)

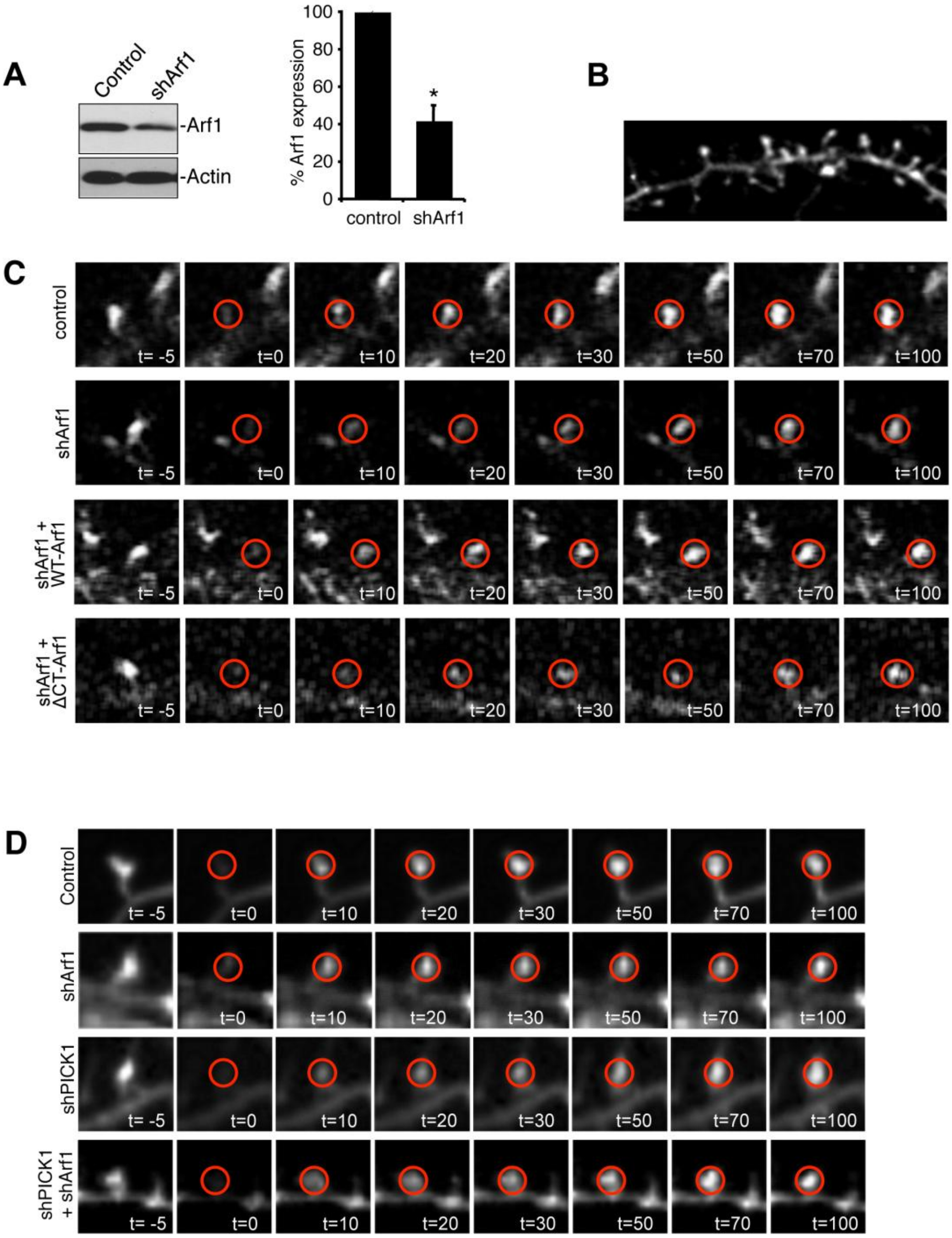

Figure S3 (Related to Figure 3)

A) Knockdown of endogenous Arf1. Cultured neurons were transfected at DIV2 with pSUPER-based shRNA against Arf1 1 or pSUPER control plasmid. 8 days after transfection neurons were harvested in lysis buffer and prepared for subsequent immunoblotting. Quantification shows a 60% reduction in endogenous Arf1 expression. Graph shows quantification of Arf1 expression (n=3), * $p<0.02$. Values are mean ± SEM.

B) Lifeact-GFP localizes to dendritic spine heads in hippocampal neurons. Dissociated hippocampal neurons were transfected with Lifeact-GFP and imaged live by confocal microscopy.

C) Representative image series for FRAP experiments corresponding to Figure 3D and E. Red circle represents bleaching area and ROI for image analysis. Time units are seconds.

D) Representative image series for FRAP experiments corresponding to Figure 3F and G. Red circle represents bleaching area and ROI for image analysis. Time units are seconds.

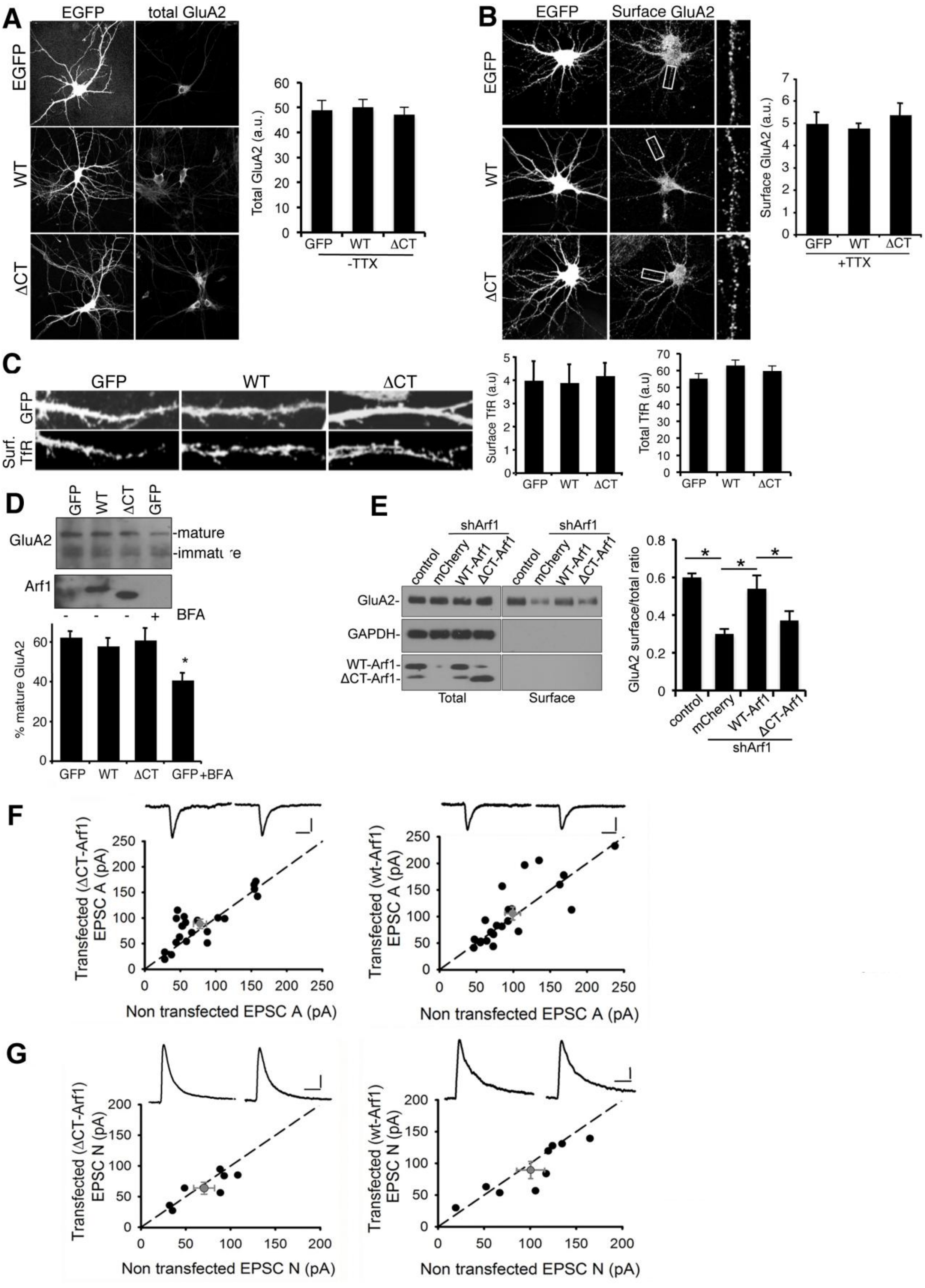
A
EGFP
total GluA2
EGFP
WT
ΔCT
Total GluA2 (a.u.)
GFP WT ΔCT
-TTX
B
EGFP
Surface GluA2
EGFP
WT
ΔCT
Surface GluA2 (a.u.)
GFP WT ΔCT
+TTX
C
GFP WT ΔCT
GFP
Surf. TfR
Surface TfR (a.u)
Total TfR (a.u)
GFP WT ΔCT
D
GFP WT ΔCT GFP
GluA2
-mature
-immature
Arf1
BFA
% mature GluA2
GFP WT ΔCT GFP+BFA
E
shArf1
control mCherry WT-Arf1 ΔCT-Arf1
GluA2-
GAPDH-
WT-Arf1-
ΔCT-Arf1-
Total
Surface
GluA2 surface/total ratio
control mCherry WT-Arf1 ΔCT-Arf1
shArf1
F
Transfected (ΔCT-Arf1) EPSC A (pA)
Non transfected EPSC A (pA)
Transfected (wt-Arf1) EPSC A (pA)
Non transfected EPSC A (pA)
G
Transfected (ΔCT-Arf1) EPSC N (pA)
Non transfected EPSC N (pA)
Transfected (wt-Arf1) EPSC N (pA)
Non transfected EPSC N (pA)

Figure S4 (Related to Figure 4)

A) Total levels of GluA2 are unaffected by overexpression of ΔCT-Arf1 or WT-Arf1 in neurons. Cells were transfected with either WT-Arf1-IRES-EGFP, ΔCT-Arf1-IRES-EGFP or empty-IRES-EGFP. Cells were fixed and subsequently stained for GluA2 under permeabilised conditions using anti-GluA2. Representative images are shown for all conditions and graph shows fluorescence intensity of GluA2 staining (n=24-26 cells, from 3 independent experiments). Values are mean ± SEM.

B) ΔCT-Arf1 overexpression has no effect on GluA2 surface expression when synaptic activity is blocked using TTX. Dissociated hippocampal neurons were transfected with either WT-Arf1-IRES-EGFP, ΔCT-Arf1-IRES-EGFP or empty-IRES-EGFP. Cells were preincubated for 1 h in the presence of 1 μM TTX, fixed and subsequently stained for surface GluA2. Representative images are shown for all conditions and graph shows fluorescence intensity of GluA2 surface staining (n=18 cells, from 3 independent experiments). Values are mean ± SEM.

C) Transferrin receptor surface levels are unaffected by overexpression of ΔCT-Arf1 or WT-Arf1 in neurons. Cells were transfected with either WT-Arf1-IRES-EGFP, ΔCT-Arf1-IRES-EGFP or control-IRES-EGFP. Cells were fixed and subsequently stained for transferrin using anti-transferrin receptor antibodies. Graph shows quantification of transferrin receptor surface staining (n=15). Values are mean ± SEM.

D) Arf1-PICK1 interactions do not regulate GluA2 ER-exit in neurons. Cultured neurons were transfected at DIV2 (in order to achieve a high transfection efficiency) with either WT-Arf1-IRES-EGFP, ΔCT-Arf1-IRES-EGFP or control-IRES-EGFP. One week later, as a positive control, one set of GFP-expressing cultures was treated with brefeldin A for 6 hours to inhibit ER exit. Cells were then lysed and extracts were subjected to EndoH digestion for 16 hours at 37°C. Proteins were detected by immunoblotting. Graph shows quantification of the percentage of GluA2 in the mature form (n=4), *$p<0.05$. Values are mean ± SEM.

E) Arf1 knockdown reduces GluA2 surface expression, which is rescued by WT-Arf1, but not ΔCT-Arf1. Hippocampal neurons were transduced with lentivirus expressing Arf1 shRNA and either mCherry alone, mCherry-IRES-sh-resistant WT-Arf1 or mCherry-IRES-sh-resistant ΔCT-Arf1, and subjected to surface biotinylation. Surface and total protein was detected by western blotting. Graph shows surface:total ratios for GluA2 (n=4), *$p<0.05$. Values are mean ± SEM.

F) AMPAR-mediated EPSCs (EPSCA) were recorded in transfected cells and nearby non-transfected cells.

Peak amplitudes (at -70 mV) were plotted for each pair (black circles). Gray symbols represent mean ± sem. Insets show representative traces (left: transfected, right: non-transfected cell). Calibration bars for all traces shown: 40 pA/20 ms.

G) NMDAR-mediated EPSCs (EPSCN) were recorded in cells transfected and nearby non-transfected cells. The amplitude measured 60 ms poststimulation (at +40 mV) were plotted for each pair (black circles). Gray symbols represent mean ± sem. Insets show representative traces (left: transfected, right: non transfected cell). Calibration bars for all traces shown: 60 pA/50 ms.

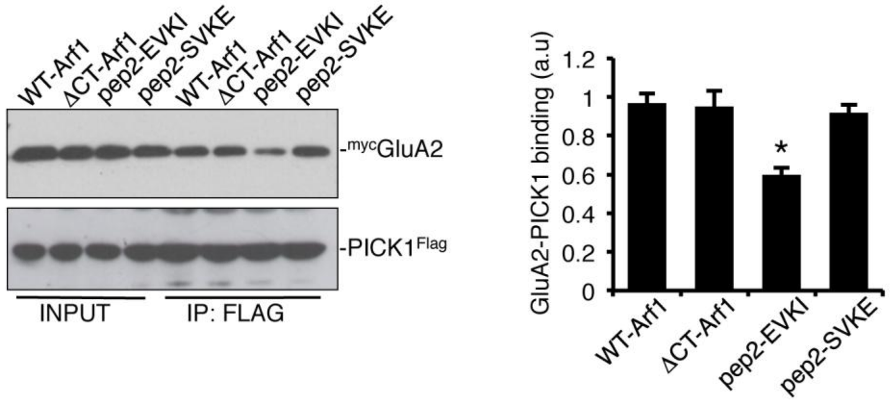

Figure S5 (Related to Figure 5)

Arf1 does not interfere with PICK1-GluA2 interaction.

COS7 cell extracts expressing PICK1^{flag} and ^{myc}GluA2 in addition to either WT-Arf1, ΔCT-Arf1 or GluA2-C-terminal peptides (active peptide pep2-EVKI, control peptide pep2-SVKE) were immunoprecipitated with anti-FLAG or IgG antibodies and bound proteins were detected by immunoblotting. Pep2-EVKI inhibits the PICK1-GluA2 interaction, whereas pep2-SVKE, WT-Arf1 and ΔCT-Arf1 have no effect. Values are mean ± SEM.

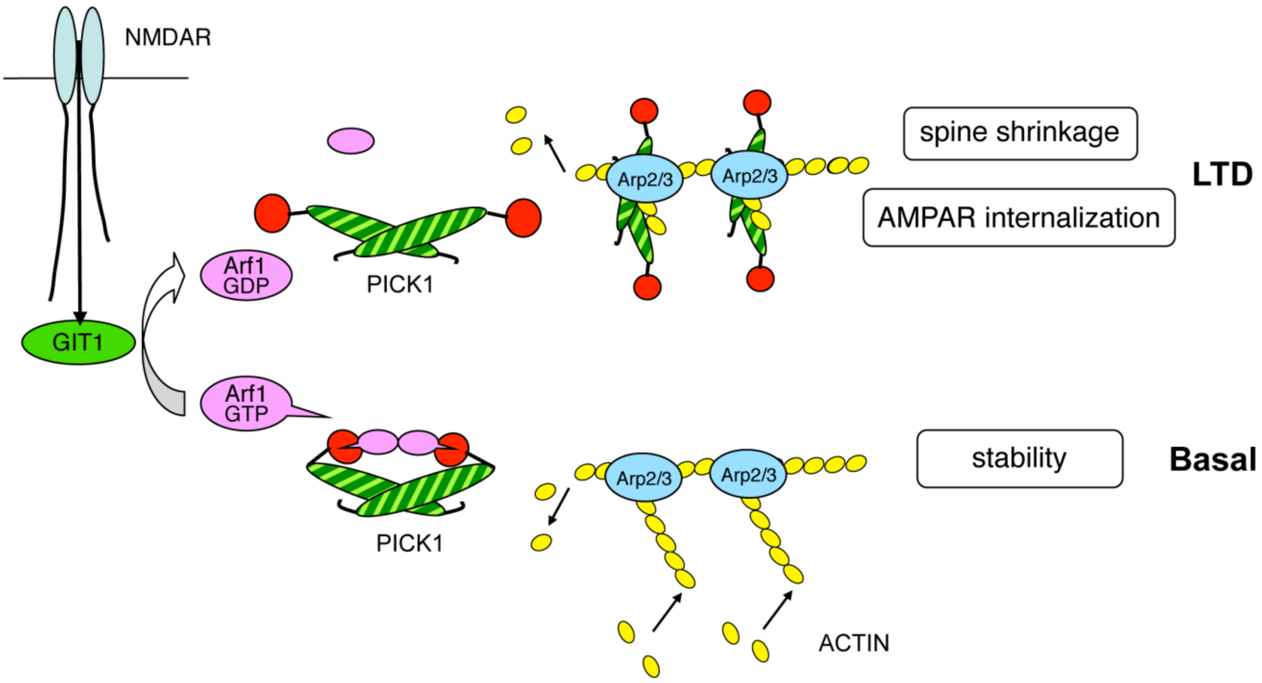

Figure S6 (Related to Figure 8)

Summary diagram representing relevant Arf1-PICK1 signaling pathways and their regulation during LTD.

Under basal conditions, a large proportion of Arf1 is in its GTP-bound state. Arf1-GTP binds PICK1 PDZ domain (red circle), and locks PICK1 in a "closed" conformation, possibly via Arf1 dimerization as shown. This results in only a weak interaction of PICK1with the Arp2/3 complex, and therefore only a low level of inhibition of Arp2/3 -mediated actin polymerization. During LTD induction, activation of NMDARs leads to stimulation of GIT1 ArfGAP activity, causing a shift towards GDP-bound Arf1. Arf1-GDP does not bind PICK1, allowing PICK1 to adopt an "open" conformation and therefore to bind more strongly to the Arp2/3 complex. PICK1 inhibits Arp2/3 activity leading to spine shrinkage and AMPAR internalization.

Supplemental Experimental Procedures

Plasmids and plasmid construction

His_6 proteins were expressed from pET28 (Novagen), GST-fusions from pGEX4T-1 (Pharmacia). For COS7 cell experiments, FLAG-tagged WT- and KD27,28AA-PICK1 were expressed from pcDNA3.1. HA-tagged WT-Arf1, Q71L-Arf1, T31N-Arf1 and GST-VHS-GAT were gifts from Prof. R. Kahn. Myc-tagged T27N-Arf6 and Q67L were gifts from Dr. T. Bouchet. Myc-Arf1 was expressed from pcDNA3.1.

For neuronal experiments, untagged WT- and ΔCT-Arf11 were expressed from pIRES-EGFP. Arf1 shRNA was expressed from a pSUPER-based plasmid kindly donated by Prof. R. Kahn as previously described (Volpicelli-Daley et al., 2005). The control construct did not contain shRNA. For rescue experiments, untagged WT-Arf1 and ΔCT–Arf1 with silent mutations: TGG AAA CgG TaG AGT ACA (mutations in lower case) were expressed from pIRES-EGFP. Lifeact-GFP was expressed from a modified pFIV vector (System Biosciences) that also expressed Arf1 shRNA and sh-resistant Arf1. Lentivirus was prepared from pFIV plasmids encoding Arf1 shRNA, mCherry and sh-resistant Arf1, and helper vectors p34N, pVSV-G.

Preparation of recombinant proteins

His_6- and GST-fusions were expressed and purified essentially as described (Rocca et al., 2008). His_6PICK1 proteins were eluted in 10 mM Tris pH7.5, 120 mM NaCl, 2 mM DTT, 200 mM imidazole. For his_6Arf1 proteins, bacteria were lysed and proteins bound to Ni^{2+}NTA beads. Following washing, bound his_6Arf1 was eluted in 8 M urea with 10 mM Tris-HCl, 100 mM Na_2PO_4 (pH 6) and 200 mM imidazole. Eluted his_6Arf1 was renatured by sequential dialysis against the same buffer with 4 M urea at pH 8, then again with 2 M urea at pH8 and finally against 10 mM Tris pH 7.4, 120 mM NaCl. Purified his_6Arf1 was then bound to either 200 μM non-hydrolysable GTP or GDP.

Antibodies

The antibodies used were as follows: Arf1 (abcam); Arf6 (Santa Cruz); Arp2/3 complex (anti-p34, BD biosciences); β-actin (Sigma); FLAG (M2, Sigma); PICK1 (goat, Santa Cruz; mouse, NeuroMab; rabbit Abcam); PSD95 (NeuroMab); GluA2 (Millipore); Synaptotagmin (BD biosciences); myc (Santa Cruz); HA

(Sigma); Rab11 (BD); Homer (Synaptic Systems); giantin (Abcam); calreticulin (Abcam); GIT1 (NeuroMab).

Buffers

Lysis Buffer: 25 mM HEPES pH 7.5, 125 mM NaCl, 0.5% TX-100, protease inhibitor cocktail (Roche).

Buffer A: 10 mM Tris pH 7.5, 0.2 mM ATP, 0.5 mM DTT, 0.2 mM $CaCl_2$.

Polymerization buffer: 50 mM KCl, 10mM Tris pH 7.5, 2 mM $MgCl_2$, 0.2 mM ATP, 0.2 mM DTT.

XB buffer: 10 mM HEPES pH 7.5, 100 mM KCl, 1 mM $MgCl_2$, 0.1 mM EDTA, 0.1% Tween-20.

HBS: 20 mM HEPES pH 7.4 (Tris), 140 mM NaCl, 5 mM KCl, 1.8 mM $CaCl_2$, 0.8 mM $MgCl_2$, 9% D-glucose.

GST pull-down assays

Pulldown assays were conducted as described (Rocca et al., 2008). Briefly, GST or GST-PICK1 was immobilised on glutathione-agarose beads in lysis buffer for 30min at 4°C. After washing, beads were incubated with 100 nM GDP- or GTP-loaded his_6Arf1 as indicated. For Arp2/3 experiments, immobilised GST or GST-PICK1 was incubated with GTP/GDP-loaded his_6Arf1 and 10 nM purified Arp2/3 complex (Cytoskeleton) in XB buffer for 1.5 h at 4°C. Beads were subsequently washed and bound proteins detected by western blotting. For Arf1 activation assays, GST-VHS-GAT was immobilised on beads, washed and incubated with extracts (500 μg) from drug-treated cultured cortical neurons. After washing, bound proteins were analyzed by western blotting.

Subcellular fractionation

This was carried out using typical fractionation procedures as described (Rocca et al., 2008).

Quantification of SDS-PAGE and Western blots

Western blot films were scanned and analyzed using NIH Image J. Error bars are standard errors of the mean (s.e.m.), and t-tests were carried out on the data using Excel.

Surface biotinylation assay

For lentiviral production, HEK293TN cells were transfected with both pFIV (system biosciences) and helper vectors p34N and pVSV-G according the manufacturers instructions using PEI (Sigma). 48 hrs after transfection, supernatant was harvested and concentrated by ultracentrifugation. Particles were aliquoted, titered and stored at -80°C.

Primary rat hippocampal cultures were infected at DIV2 for 6hrs before being returned to conditioned media. Around 18-19 days later neurons were surface biotinylated and processed as described (Hanley and Henley, 2005).

Actin polymerization assays

Pyrene-labelled actin (Cytoskeleton) was prepared in buffer A and centrifuged at 200,000 g for 1 h at 4°C to remove actin oligomers. Polymerization reactions were carried out essentially as described (Rocca et al., 2008) using 2.5 μM pyrene-actin, 25 nM Arp2/3, 100 nM GST-VCA (N-WASP verprolin homology / cofilin / acidic domain) with or without 200 nM his_6PICK1 and 200 nM his_6Arf1 in polymerization buffer. Reactions were subsequently transferred to a cuvette and fluorescence data collected in a Spex Fluoromax fluorimeter at an emission wavelength of 386nm at 26°C. Kinetic analysis was performed by calculating the rate of actin assembly as a function of fluorescence against time taken to reach 50% maximal polymerization. Pyrene-labeled actin, GST-WASP VCA and Arp2/3 complex were from Cytoskeleton Inc.
